# Machine-Learning-Driven Performance Optimization of Nano-Modified Construction Materials: A Bibliometric Analysis and Systematic Review

**DOI:** 10.3390/nano16191212

**Published:** 2026-09-24

**Authors:** Xiaochuan Liu, Gaofei Kong, Jiaji Hu, Qiqi Zheng, Xingqiang Li, Haijie He, Jing Yu

**Affiliations:** 1 School of Architectural Engineering, Yan’an University, Yan’an 716000, China; 2 Yan’an Medical College, Yan’an University, Yan’an 716000, China; 3 School of Civil Engineering and Architecture, Taizhou University, Taizhou 318000, China; 4 College of Civil and Architectural Engineering, Zhejiang University, Hangzhou 310058, China

**Keywords:** nano-modified construction materials, machine learning, explainable artificial intelligence, performance prediction, multi-objective optimization, bibliometric analysis, systematic review

## Abstract

Nanomaterials improve the fresh-state behavior, mechanical performance, durability, and multifunctionality of construction materials through packing and filling, nucleation, interfacial regulation, crack bridging, transport-barrier effects, and functional responses. These benefits depend on coupled material, processing, matrix, and service variables, making conventional trial-and-error development inefficient. Machine learning (ML) offers a data-driven route for resolving nonlinear composition–processing–structure–property relationships. This review combines a CiteSpace-based bibliometric analysis of Web of Science Core Collection (WoSCC) records with a structured qualitative synthesis of ML-assisted studies on zero-dimensional nanoparticles, one-dimensional high-aspect-ratio nanomaterials, two-dimensional layered nanomaterials, functional nanomaterials, carbon dots, and emerging hybrid systems. Annual output increased from four publications in 2012 to 268 in 2025, and ML showed the strongest recent keyword burst (strength = 22.57). Across representative studies, reported test-set coefficients of determination commonly ranged from 0.858 to 0.995, although validation designs and data independence varied markedly. Tree-based ensembles were generally effective for small-to-medium tabular datasets, whereas deep learning was most defensible for images, spectra, and time-series data. Prediction estimates properties for a specified formulation; optimization uses a validated surrogate model to search the formulation or processing space and therefore requires independent experimental verification. Nano-SiO_2_, carbon nanotubes, and graphene oxide dominate the evidence base, while durability, microstructure, uncertainty quantification, and multifunctional co-optimization remain underrepresented. Matrix-aware standardized databases, physically constrained models, grouped or external validation, and closed-loop experiments are required for reliable intelligent design. The bibliometric component covers 1324 Web of Science Core Collection records published during 2012–2026, whereas the qualitative component applies explicit criteria for construction relevance, definition of the ML task, and sufficiency of performance and validation reporting. Bibliometric patterns and qualitative evidence are therefore reported and interpreted separately.

## 1. Introduction

Construction materials encompass cementitious materials, asphalt, functional coatings, and a broad range of composites used in building envelopes and thermal-management systems. Their performance directly affects structural safety, durability, and service functionality. These material classes differ substantially. Cementitious materials are multiphase, porous, and heterogeneous across length scales, and their macroscopic behavior is jointly governed by raw-material composition, hydration, the interfacial transition zone, pore structure, and crack evolution. Asphalt exhibits pronounced temperature sensitivity and viscoelasticity, whereas functional coatings are strongly affected by interfacial adhesion, penetrant transport, and environmental aging. As engineering structures are increasingly required to provide high strength, toughness, durable protection, and intelligent responses, conventional formulations and processing routes often fail to satisfy multiple objectives simultaneously. Nanoscale regulation of matrix structure, interfacial behavior, and transport processes has therefore become an important strategy for improving the overall performance of construction materials [1,2,3].

Nanomaterials provide new routes for performance regulation because of their small particle size, high specific surface area, and pronounced interfacial activity. According to dimensionality and primary function, they may be classified as zero-dimensional particles, such as nano-SiO_2_, nano-Al_2_O_3_, and nano-CaCO_3_; one-dimensional high-aspect-ratio materials, such as carbon nanotubes (CNTs), carbon nanofibers (CNFs), cellulose nanofibrils (CeNFs), and cellulose nanocrystals (CNCs); two-dimensional layered materials, such as graphene oxide (GO), hexagonal boron nitride (h-BN), molybdenum disulfide (MoS_2_), and layered double hydroxides (LDHs); and functional or emerging nanomaterials, such as nano-TiO_2_, nano-ZnO, nano-encapsulated phase-change materials (nano-PCMs), and carbon dots (CDs) [3,4]. These materials act through particle packing and filling, heterogeneous nucleation, pozzolanic reaction, crack bridging, interfacial reinforcement, transport-barrier effects, and photo- or thermoresponsive mechanisms. Their specific effects depend on the chemical environment and structure of the host matrix, whether cementitious, asphaltic, or coating-based. Beyond construction, nanoscale control is also used in areas such as targeted drug delivery, illustrating the broad transferability of size-, surface-, and interface-dependent design principles [5]; the present review, however, applies these principles specifically to construction materials.

In cementitious systems, nano-SiO_2_ combines microfilling, heterogeneous nucleation, and pozzolanic activity. It promotes the formation of calcium silicate hydrate (C-S-H), refines the pore structure and interfacial transition zone (ITZ), and thereby improves mechanical performance and durability [6,7,8,9]. Other oxide and mineral nanoparticles, including nano-TiO_2_, nano-Fe_2_O_3_, nano-CaCO_3_, and nanoclay, can also regulate strength development and volumetric stability through filling, nucleation, or chemical reactivity [10]. CNTs can transfer load, bridge cracks, and suppress crack propagation [11,12], while CNT-based cement composites can also provide electrical and self-heating functions [13]. Cellulose nanofibrils (CeNFs), cellulose nanocrystals (CNCs), and carbon nanofibers (CNFs) provide complementary crack-bridging and microstructural reinforcement effects [14,15,16,17,18], whereas two-dimensional materials such as GO can regulate hydrate growth, strengthen interfaces, and increase the tortuosity of transport pathways for aggressive species [19,20]. Across other construction-material systems, nano-TiO_2_ has been used in photocatalytic and self-cleaning coatings [21], while nano-ZnO can improve resistance to photo-oxidative aging in asphalt [22]. Encapsulated phase-change materials provide thermal regulation in cementitious components and building envelopes [23]. CNTs, GO, and hybrid nanosystems also show potential for asphalt toughening, barrier protection, and integrated structural–functional performance.

The effectiveness of nano-modification is not determined solely by the material type and dosage. It is jointly affected by particle size, morphology, surface state, dispersion quality, matrix composition, processing route, and service environment [2,3]. GO can agglomerate in alkaline cement pore solution because of ionic interactions [24]; CNT dispersion likewise depends strongly on dispersants and mixing procedures [11,12,25,26], whereas excessive nano-SiO_2_ may increase water demand and reduce workability [27]. In asphalt and coating systems, compatibility with the organic matrix, interfacial adhesion, and thermo-oxidative aging further alter the reinforcement efficiency. Poor dispersion or an unsuitable dosage can convert agglomerates into local defects and stress concentrators. Relationships between nanomaterial descriptors and construction-material performance are therefore nonlinear and matrix dependent, making the optimum dosages and processing conditions reported by different studies difficult to compare directly.

The conventional development of nano-modified construction materials relies primarily on controlled-variable experiments and iterative mixture trials. The number of possible experiments increases rapidly when nanomaterial type, size, dosage, and surface state vary simultaneously with matrix composition, dispersant, ultrasonication parameters, mixing procedure, and curing or aging regime. Exhaustive testing is costly and time-consuming and is sensitive to differences in raw materials, testing standards, and sample size. In cementitious materials, composition–processing–structure–property relationships span hydration, interfaces, pores, and crack evolution, and conventional empirical equations or statistical regressions cannot adequately represent the nonlinear coupling among high-dimensional variables [28,29]. Asphalt, coating, and thermal-management composites additionally require consideration of temperature, irradiation, cyclic loading, and interfacial aging. Strong interactions across all these systems make one-factor-at-a-time experiments inadequate for identifying synergistic, competitive, or antagonistic effects.

Machine learning (ML) provides a data-driven paradigm for addressing these limitations. Algorithms such as artificial neural networks (ANNs), support vector machines (SVMs), random forests (RFs), extreme gradient boosting (XGBoost), and deep learning (DL) can learn nonlinear mappings between input descriptors and target properties from experiments, published literature, numerical simulations, and microstructural characterization. ML has been widely applied to predict the mechanical properties of concrete [30]. Broader cementitious-material reviews also document applications to workability, durability, transport-related properties, and life-cycle performance [28,31], while ML has expanded to the rheology and aging of asphalt, the structural and protective performance of functional coatings, and heat-transfer responses of building thermal-management materials. Coupling ML with genetic algorithms (GAs), particle swarm optimization (PSO), Bayesian optimization (BO), or other multi-objective methods enables inverse searches for compositions and processing parameters. Shapley additive explanations (SHAP), feature-importance analysis, and partial dependence plots (PDPs) can further identify influential variables, thresholds, and interactions, extending ML from property fitting toward mechanistic interpretation and materials optimization [31].

Machine learning is particularly well suited to the multivariable coupling encountered in nano-modified construction materials. Yang et al. developed a multimodal ML model for CNT- and GO-reinforced cementitious composites and obtained a coefficient of determination (R^2^) of 0.96 for 28-day compressive strength. SHAP analysis further identified favorable ranges of CNT dosage and length and of GO sheet size and layer number [32]. Related investigations have addressed nano-SiO_2_-modified concrete [6], graphene-nanoplatelet-reinforced cementitious composites [33], CNT-reinforced cementitious composites [34,35,36], and the high-temperature performance of GO-modified materials [37]. Studies on aging prediction for nano-ZnO/styrene-butadiene rubber (SBR)-modified asphalt, structural inversion of porous TiO_2_ coatings, and thermal-response modeling of phase-change materials indicate that analogous data-driven workflows can be extended to other construction materials. Nevertheless, feature meanings and governing mechanisms differ among matrices, so transferred models require recalibration and experimental validation.

**Figure 1:**
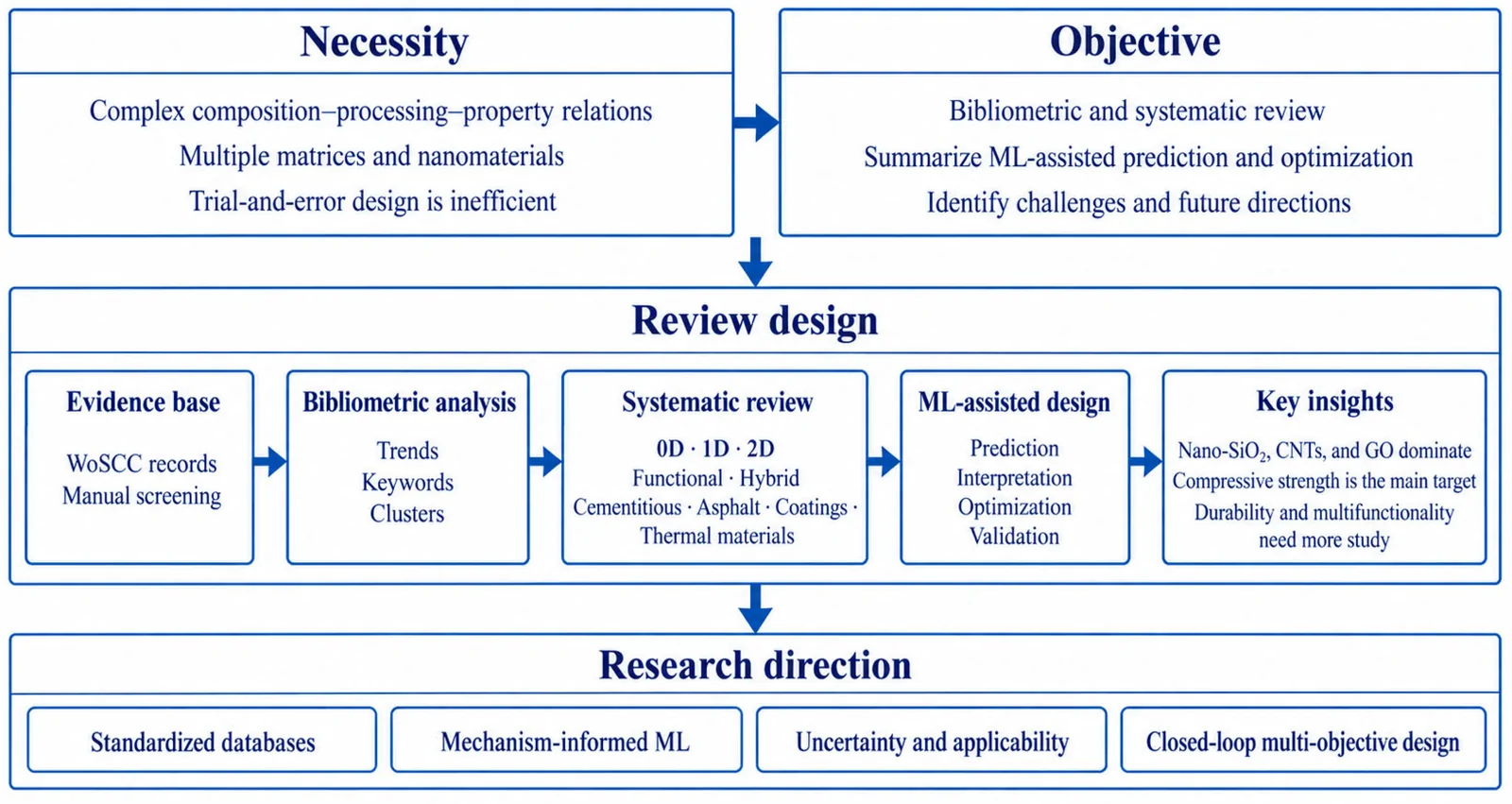
Scope and analytical framework of the review, integrating bibliometric mapping and systematic evidence synthesis with ML-assisted analysis and future research directions.

Despite rapid growth at this interdisciplinary frontier, existing reviews generally address either nanomaterial reinforcement and dispersion or ML prediction for construction materials, rather than systematically linking nanomaterial descriptors, matrix type, governing mechanism, data structure, learning method, and target property [2,3,4,28,29]. This review therefore examines ML-driven performance optimization of nano-modified construction materials. First, a CiteSpace-based bibliometric analysis of WoSCC records is used to reveal publication trends, the keyword co-occurrence and cluster structure, and the evolution of burst terms. Second, ML-assisted prediction and optimization are reviewed according to nanomaterial dimensionality and function: zero-dimensional nanoparticles, one-dimensional high-aspect-ratio nanomaterials, two-dimensional layered nanomaterials, functional nanomaterials, and CDs and emerging hybrid systems. Cementitious materials, asphalt, functional coatings, and building thermal-management materials are compared in terms of data availability, feature representation, modeling tasks, and evidential boundaries. Finally, challenges and future directions are discussed with respect to data quality, cross-matrix generalization, interpretability, integration of physical mechanisms, multi-objective optimization, and engineering validation. The resulting framework is intended to support property prediction, formulation design, and intelligent optimization across nano-modified construction materials. Figure 1 summarizes the relationship between the evidence base, bibliometric mapping, systematic synthesis, ML workflow, and material-design outcomes.

Accordingly, the review addresses four questions: (1) Which nanomaterials, construction-material matrices, and target properties dominate ML-assisted research? (2) Which algorithms and validation strategies provide the most credible evidence? (3) Which nanomaterial and processing descriptors are required for physically meaningful models? (4) Which barriers impede the transition from property prediction to experimentally validated, closed-loop optimization?

## 2. Bibliometric Analysis

### 2.1. Data Sources and Research Methods

A quantitative bibliometric analysis was conducted to characterize the research status, knowledge structure, and evolution of ML-driven performance optimization in nano-modified construction materials. The Web of Science Core Collection (WoSCC) was selected because of the completeness of its bibliographic, abstract, and citation metadata, and relevant records were retrieved using a topic search (TS). The WoSCC search was executed on 10 August 2026. The search period extended from January 2012 to July 2026, covering the transition of this interdisciplinary field from initial exploration to rapid growth; records from 2026 therefore reflect database coverage available on the retrieval date. Only English-language research articles were retained to minimize the effects of reviews and conference records on keyword frequencies and citation-burst results. The query included the terms cement, concrete, cementitious, mortar, construction material, asphalt, pavement, and composite to capture studies on cementitious materials, asphalt, functional coatings, and other construction composites and to permit comparisons among matrices. WoSCC was used as the sole source because its standardized citation and cited-reference fields are directly compatible with CiteSpace; this choice improves metadata consistency but may omit relevant studies indexed only in other databases.

TS = ((“machine learning” OR “deep learning” OR “neural network*” OR “artificial intelligence” OR “random forest” OR “support vector machine” OR “XGBoost” OR “ensemble learning” OR “transfer learning” OR “reinforcement learning” OR “generative adversarial network” OR “CNN” OR “convolutional neural network” OR “ANN” OR “artificial neural network”) AND (“nano-silica” OR “nano-alumina” OR “nano-MgO” OR “nano-ZrO_2_” OR “nano-Fe_2_O_3_” OR “nano-CaCO_3_” OR “nano-clay” OR “carbon nanotube*” OR “CNTs” OR “nanocellulose” OR “CNFs” OR “graphene oxide” OR “GO” OR “hexagonal boron nitride” OR “h-BN” OR “nano-MoS_2_” OR “layered double hydroxide*” OR “LDH” OR “nano-TiO_2_” OR “nano-ZnO” OR “nano-PCM” OR “carbon dots” OR “carbon quantum dots”) AND (“cement*” OR “concrete” OR “cementitious” OR “asphalt” OR “bitumen” OR “mortar” OR “building material*” OR “construction material*” OR “pavement” OR “composite*”)).

Records were deduplicated in CiteSpace by comparing titles, digital object identifiers (DOIs), authors, and publication years and retaining the record with the more complete metadata. Screening was then conducted in two stages. Titles and abstracts were first assessed against three mandatory concepts: an ML method, a nanoscale modifier or nanostructure, and a construction-material matrix or construction-relevant performance problem. Full texts were subsequently checked where relevance remained uncertain. Records on automotive, aerospace, biomedical, electronic, or generic polymer composites were excluded unless a construction matrix or a directly transferable construction-material task was explicit. This rule was applied particularly to the broad term “composite”; because the search blocks were connected by AND, the term could not retrieve a record by itself, and manual screening removed residual off-scope records. After deduplication and screening, 1324 articles were retained for bibliometric mapping.

The qualitative synthesis used the screened bibliometric corpus as its sampling frame but applied additional eligibility criteria. A study was included in the detailed synthesis when it (i) reported a nano-modified construction material or a clearly delimited transferable workflow, (ii) specified the ML task and input–output variables, and (iii) provided sufficient information on dataset size, validation, performance, interpretation, optimization, or experimental verification to support critical assessment. Bibliometric indicators were calculated for the complete screened corpus, whereas the qualitative synthesis focused on representative studies meeting these reporting criteria. The detailed qualitative synthesis comprised 82 unique publications, defined as the publications cited and critically discussed in Section 3. A structured subset of the representative studies is presented in Section 3 and is not intended as the full inclusion list. The workflow follows the logic of Preferred Reporting Items for Systematic Reviews and Meta-Analyses (PRISMA): identification, deduplication, title/abstract screening, full-text eligibility assessment, and inclusion. The search string, database, period, document and language filters, screening rules, and analytical settings are reported here to permit replication; the WoSCC search was executed on 10 August 2026. The query and publication-year window are provided, but the dataset cannot be recreated as an identical time-stamped snapshot, particularly for the incomplete 2026 publication year.

Methodological quality was assessed across six domains: dataset provenance and size; completeness of material and processing descriptors; separation of training, validation, and test data; use of grouped or external validation; reporting of error metrics and uncertainty; and independent experimental verification of optimized candidates. “Prediction” denotes estimation of a response for specified inputs. “Optimization” denotes a search over admissible inputs using a validated predictive model and explicit objective functions or constraints. Model ranking was therefore not based on R2 alone, and optimization claims were considered stronger only when recommended formulations or processing conditions were experimentally verified. During extraction, reported metrics were assigned to training, internal validation, cross-validation, hold-out testing, or external testing only when the original article identified the relevant subset; otherwise, the validation status was recorded as not reported. Potential leakage was evaluated from the reported splitting and preprocessing procedures, but it was not inferred when methodological details were insufficient. Dosage units were retained with their original denominators when conversion would alter their physical meaning.

CiteSpace was used as the primary visualization tool to construct and analyze the domain knowledge map. Annual publication trends, keyword co-occurrence, clustering, and keyword bursts were examined to identify major themes and evolving frontiers in ML applications to nano-modified cementitious materials, GO, and CNT-reinforced composites. The time slice was set to one year, and networks were pruned using the Pathfinder algorithm. For keyword analysis, nodes in the top 10% by frequency were retained for each time slice, with no more than 100 nodes per slice, balancing information coverage and graphical clarity [38,39]. These settings are widely used in bibliometric studies and facilitate identification of the principal structural relationships within a knowledge network.

**Figure 2:**
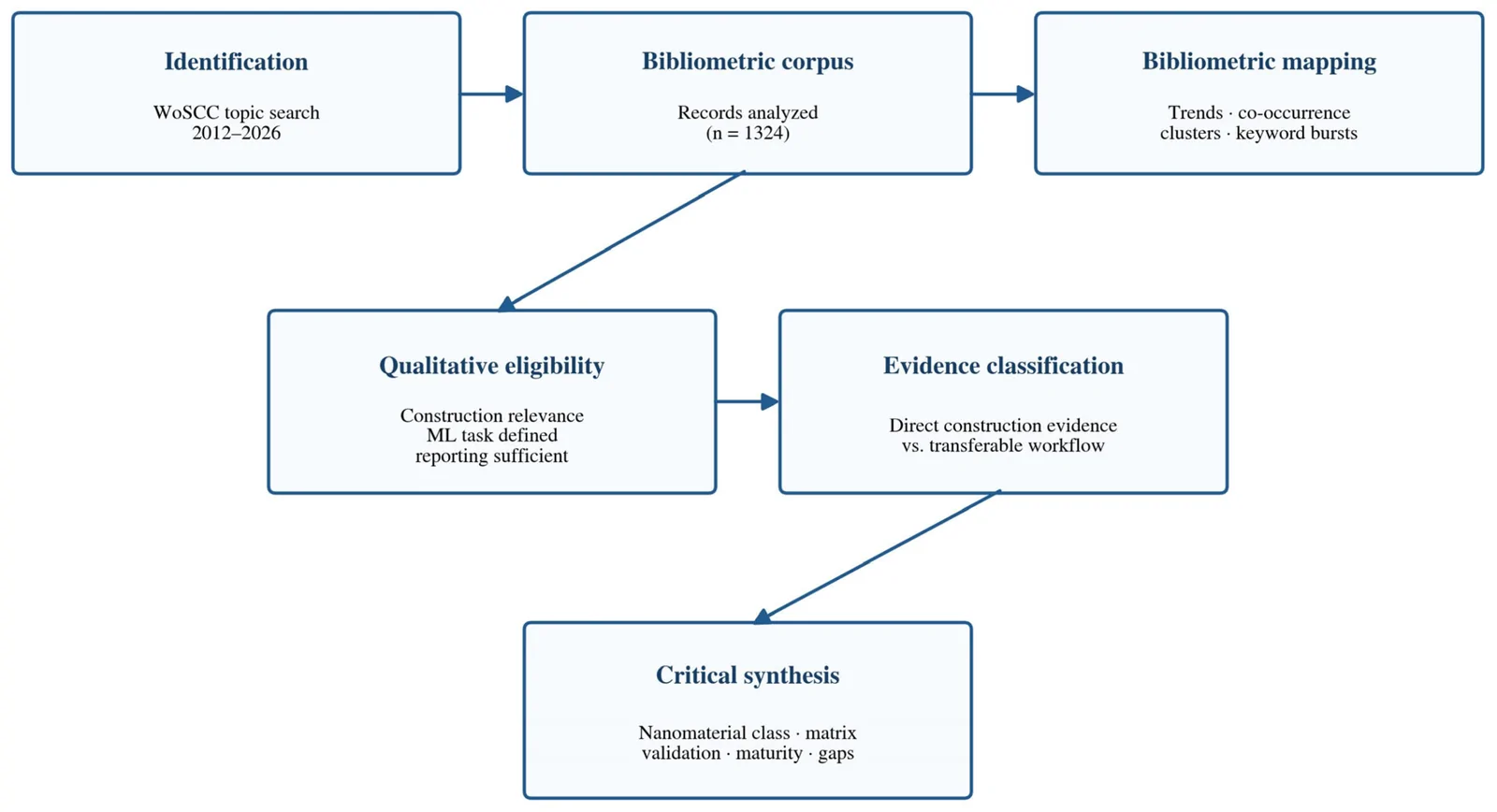
PRISMA-style overview of record identification, bibliometric mapping, and qualitative evidence selection. The final bibliometric corpus comprised 1324 records, and the detailed qualitative synthesis included 82 unique publications; stage-specific screening counts could not be reliably recovered from the available study records.

The PRISMA-style evidence-selection workflow is presented in Figure 2 immediately after the methodology. Bibliometric mapping and visualization were performed using CiteSpace version 7.0.R0. The final bibliometric corpus comprised 1324 records, of which 82 unique publications were included in the detailed qualitative synthesis. The available documentation does not establish the intermediate identification, deduplication, screening, and exclusion counts. Figure 2 therefore depicts the PRISMA-style selection logic rather than a complete PRISMA flow diagram; these gaps limit quantitative auditability.

### 2.2. Publication Trends and Growth Characteristics

**Figure 3:**
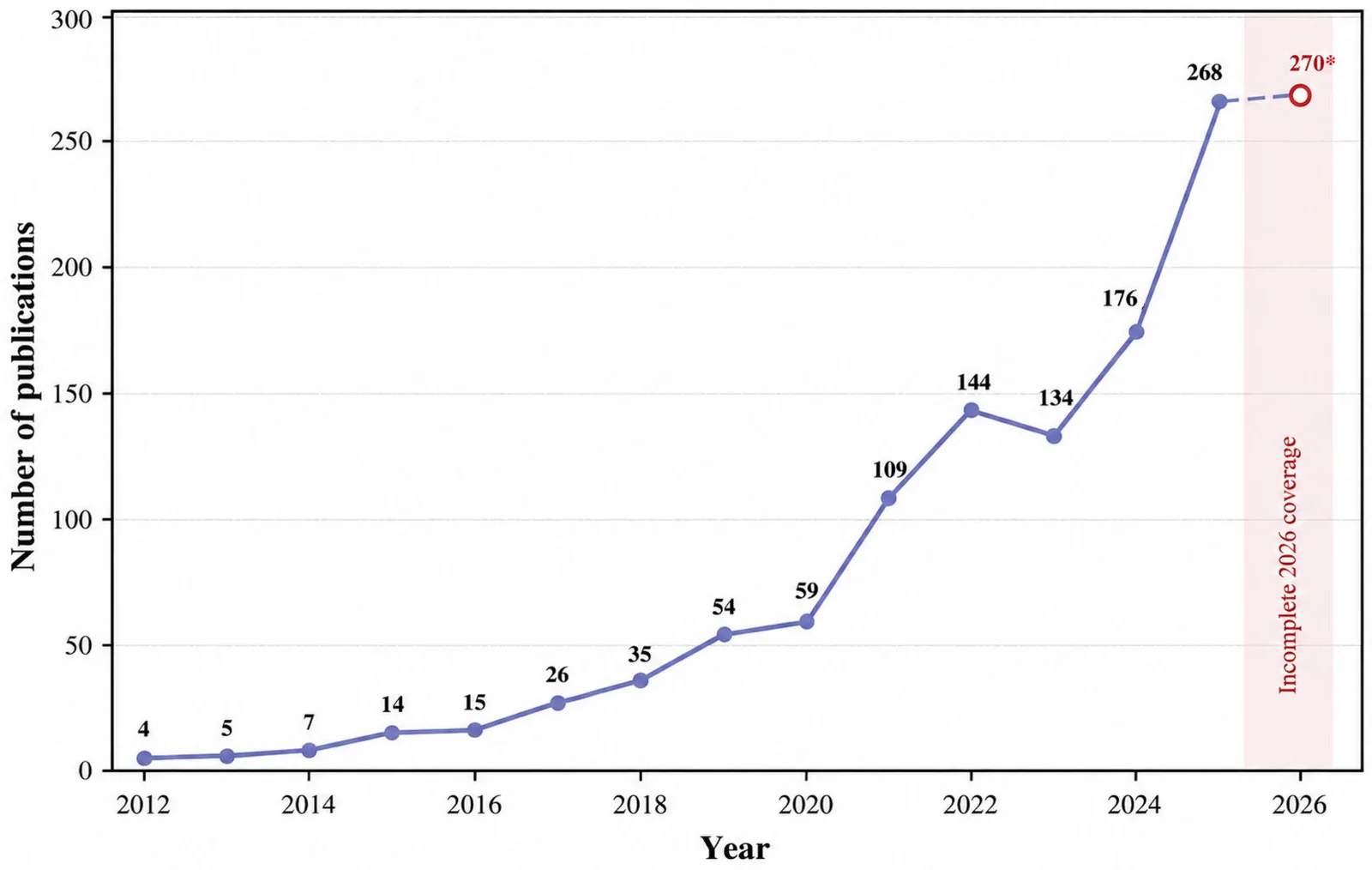
Number of publications per calendar year from January 2012 to July 2026. The vertical axis reports annual publication count, and the horizontal axis advances in one-year increments; the 2026 value reflects incomplete database coverage as of the retrieval date, 10 August 2026. * indicates that the 2026 count is based on incomplete database coverage.

As shown in Figure 3, annual publication output in this interdisciplinary field increased markedly between 2012 and 2026, from four articles in 2012 to 268 in 2025 and 270 in 2026. The topic has thus progressed from exploratory work to an active research area at the intersection of intelligent construction materials and nano-modification. Growth accelerated after 2021, reflecting increasingly extensive use of ML to predict and optimize the properties of cementitious materials, asphalt, and composites modified with nano-SiO_2_, CNTs, GO, and related nanomaterials. Because database coverage for 2026 may still be incomplete, comparisons with full calendar years should be interpreted cautiously.

Three stages can be distinguished. During the exploratory stage (2012–2016), annual output remained between four and 15 articles, and most work involved initial applications of conventional ANNs. During the early-growth stage (2017–2020), output increased from 26 to 59 articles and methods expanded from individual models to SVMs and response surface methodology (RSM). During the rapid-growth stage (2021–2026), annual publication counts reached 109, 144, 134, 176, 268, and 270, respectively. ML consequently emerged as a major approach for intelligent and data-driven optimization of nano-modified construction materials, with growing emphasis on multimodal data fusion, sensitivity analysis, and nanocomposite design. Although this interdisciplinary field developed relatively late, its rapid expansion demonstrates a strong emerging research profile.

### 2.3. Keyword Co-Occurrence and Research Clusters

**Figure 4:**
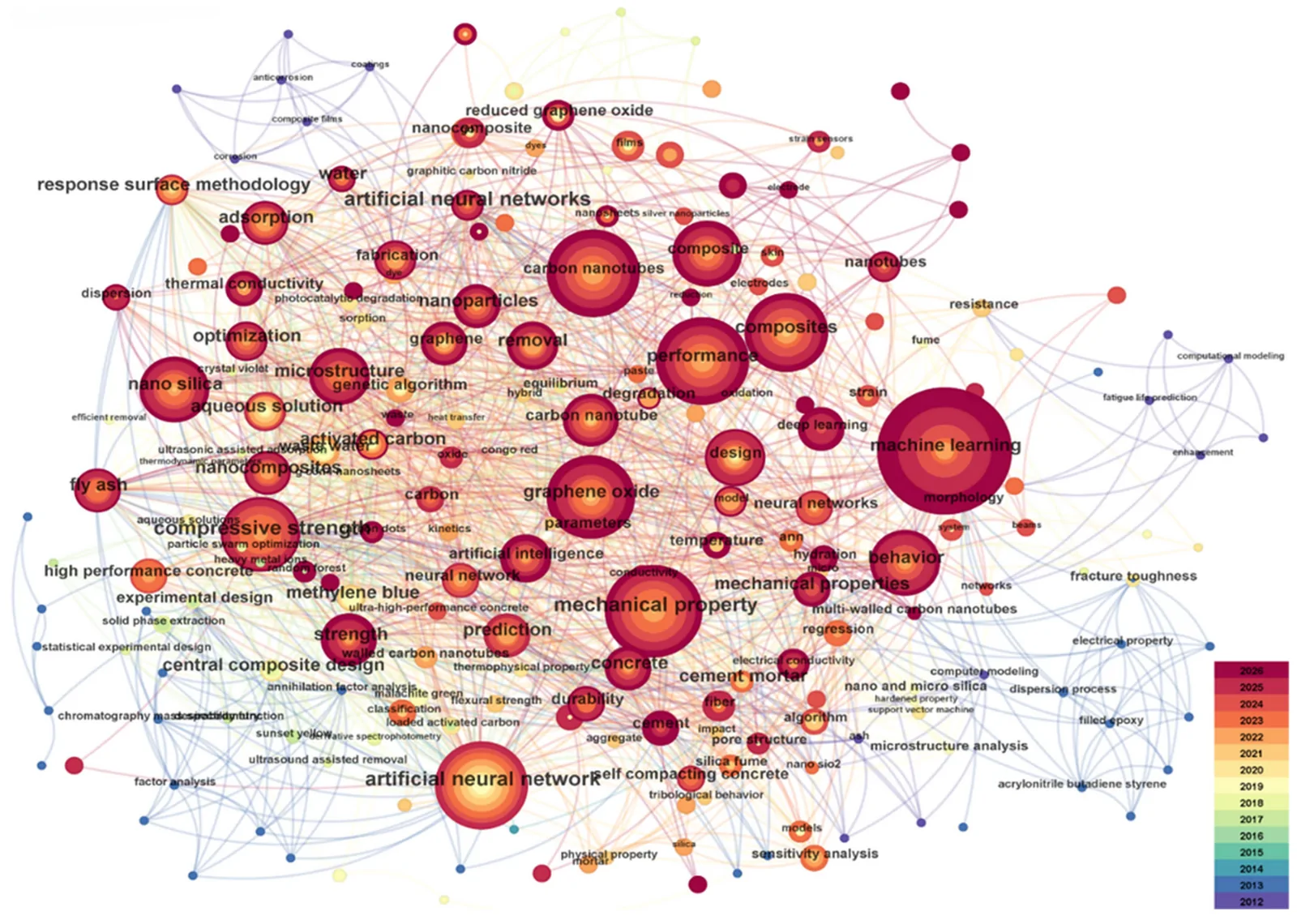
Keyword co-occurrence network.

Keyword co-occurrence analysis provides an overview of the major topics and knowledge structure of ML-driven optimization of nano-modified construction materials. As shown in Figure 4 and Table 1, the most frequent terms were machine learning (258), carbon nanotubes (184), mechanical property (172), artificial neural network (159), performance (157), graphene oxide (134), composites (131), compressive strength (115), behavior (112), and nano-silica (95). Machine learning and carbon nanotubes occupied central positions by frequency, while artificial neural network had the highest betweenness centrality (0.22), indicating a bridging role among research themes. Counts and centralities refer specifically to the screened domain of ML-enabled research involving nanomaterials and construction-related materials or performance tasks.

The keyword distribution reveals four principal directions. First, artificial neural network, machine learning, neural network, and prediction represent the development and optimization of data-driven predictive models. Second, mechanical property, compressive strength, behavior, and performance reflect the dominant focus on strength, modulus, and deformation of nano-modified cementitious materials, high-performance concrete, and related composites. Third, the high frequencies of carbon nanotubes, graphene oxide, nano-silica, and nanoparticles demonstrate the close integration of ML with major nanomodifier classes. Finally, composites, resistance, and sensitivity analysis indicate a shift beyond prediction toward composite design, durability assessment, and interpretation of influential parameters.

Overall, the co-occurrence network delineates a progression from nanomaterial incorporation through model development and property prediction to optimization. Mechanical-property prediction, the development of ANN and ML approaches, and the use of CNTs and GO are the most representative themes.

**Figure 5:**
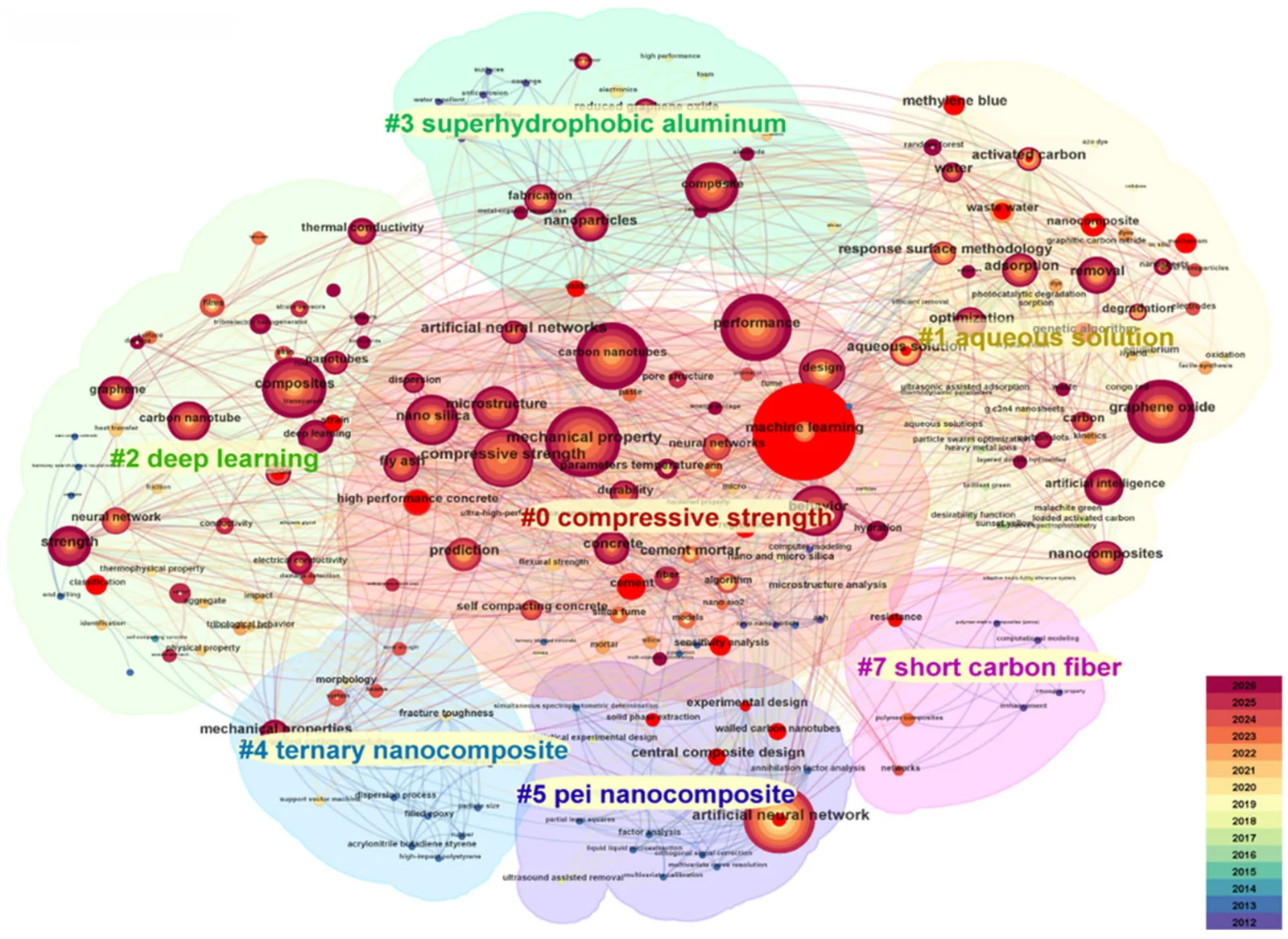
Keyword cluster map. Cluster labels beginning with “#” are CiteSpace-assigned cluster identifiers ordered by cluster size; the shaded regions distinguish keyword clusters, while node and link colors follow the temporal color scale shown in the figure.

Keyword clustering was performed on the co-occurrence network to further resolve the knowledge structure (Figure 5). Cluster labels were generated automatically from citing-record terms using the log-likelihood-ratio procedure and are treated as descriptive labels rather than unique or causal categories. The numerical modularity Q and mean silhouette S outputs are not documented in the available analysis records. Cluster separation and internal homogeneity therefore cannot be evaluated quantitatively; thematic interpretation relies on membership, network position, and construction-specific relevance and should be treated cautiously.

Cluster #0, “compressive strength,” was the largest and most central cluster and contained directly construction-related terms, including concrete, cement, high-performance concrete, durability, and prediction. Cluster #2, automatically labeled “deep learning,” joined methodological terms with carbon nanotube, graphene, composite, and strength. Cluster #1 contained aqueous-solution and adsorption terminology. Its label and interpretation are less secure because these generic terms can arise from precursor characterization or from adjacent environmental-material applications rather than from the construction matrix itself. Accordingly, clusters #1 and #2 are interpreted from their complete membership and links to construction-specific nodes, not from the automatic label alone. Their presence also exposes a residual limitation of broad interdisciplinary searches and is not taken as direct evidence of construction-material application.

Taken together, the cluster map supports a robust property-prediction core centered on concrete strength, CNTs, GO, and ML. Smaller peripheral clusters provide evidence of methodological transfer but should be interpreted cautiously. The map therefore distinguishes directly construction-focused clusters from adjacent or weakly connected topics rather than assigning equal evidential weight to all automatically generated labels.

### 2.4. Evolution of Research Frontiers

Keyword-burst analysis reveals temporal changes in research hotspots. As summarized in Table 2, the field evolved from experimental design and conventional neural networks, through deeper nanomaterial characterization and multi-algorithm integration, to ML-centered nanocomposite optimization. Several burst terms (e.g., aqueous solution, activated carbon, and model) are intentionally retained because burst detection reports database terminology; they are general or adjacent-domain signals and are not interpreted as construction-specific findings.

During the early stage (2013–2019), the principal burst terms were central composite design, experimental design, artificial neural network, response surface methodology, aqueous solution, activated carbon, and methylene blue. Research emphasized the introduction of experimental-design methods and basic ANN models and preliminary applications of nanomaterials in adsorption and solution systems, indicating a phase of methodological adoption and initial validation.

During the intermediate stage (2020–2023), burst terms shifted toward graphene oxide, resistance, sensitivity analysis, high-performance concrete, model, walled carbon nanotubes, classification, and regression. The prominence of GO and high-performance concrete indicates growing interest in GO modification and performance prediction for advanced concrete. The concurrent emergence of sensitivity analysis, classification, and regression further shows that attention moved from conceptual demonstrations toward material-property assessment, model validation, and engineering applications.

During the recent stage (2024–2026), machine learning exhibited the highest burst strength (22.57), accompanied by cement, oxide, and nanocomposite. The frontier therefore shifted from conventional neural networks and experimental design toward an integrated ML framework for cementitious nanocomposite design and optimization of oxide nanomaterials. The sustained burst of machine learning suggests that its role is progressing from auxiliary prediction toward optimization and intelligent materials design.

In summary, the research frontier has progressed from experimental design and conventional neural networks, through multi-algorithm integration and increasingly detailed nanomaterial characterization, to ML-centered intelligent optimization of nanocomposites. Mechanical-property prediction, CNT and GO modification, and integrated ML workflows now represent the most prominent frontier directions.

## 3. Machine-Learning-Assisted Performance Optimization by Nanomaterial Class

The maturity of ML applications varies substantially with nanomaterial dimensionality and the construction-material matrix. Zero-dimensional nanoparticles are supported by comparatively abundant strength and durability data, enabling workflows that combine model benchmarking, explainable analysis, and multi-objective formulation optimization. Models for one- and two-dimensional materials must supplement conventional composition variables with structural and processing descriptors such as aspect ratio, sheet size, surface functionality, and dispersion method. Data for functional nanomaterials and CDs are more fragmented and span cementitious materials, asphalt, functional coatings, and building thermal-management systems. The following sections therefore compare these nanosystems through the sequence of material characteristics, governing mechanisms, data representation, modeling task, and optimization objective, while distinguishing transferable methodologies from matrix-specific relationships and evidential limitations. Dimensionality is used only as an organizational scaffold and does not imply chemical equivalence. Pristine and functionalized CNTs differ in surface charge, dispersion, and interfacial bonding, while GO and reduced graphene oxide (rGO) differ in oxygen content, conductivity, and affinity for hydration products. Models that encode only a generic material label can therefore confound chemistry with dosage; surface functionalization, reduction degree, defect density, and dispersion protocol should be explicit descriptors. Table 3 summarizes the representative ML studies discussed in the following subsections.

### 3.1. Zero-Dimensional Nanoparticles

Zero-dimensional nanoparticles were among the earliest and most extensively investigated modifiers for cementitious materials. Representative examples include nano-SiO_2_, nano-Al_2_O_3_, nano-ZrO_2_, nano-Fe_2_O_3_, nano-CaCO_3_, and nanoclay. Their principal effects comprise pozzolanic activity, such as consumption of calcium hydroxide by nano-SiO_2_ to form additional C-S-H; nucleation, through provision of sites for hydrate precipitation and acceleration of early hydration; particle packing and filling of capillary and gel-scale voids; and densification of the ITZ. These mechanisms can jointly improve mechanical performance and durability but may reduce workability and complicate dispersion. Strong nonlinear coupling among nanoparticle dosage, water-to-binder ratio, age, curing regime, and macroscopic properties has made ML a prominent analytical tool. Existing studies encompass ANNs, RFs, XGBoost, SVMs, ensemble learning, metaheuristic optimization, and explainable artificial intelligence (XAI), particularly SHAP and PDP analysis. Compressive strength remains the dominant target, although durability, multi-objective mixture optimization, and high-temperature performance are receiving increasing attention [53]. Here, the packing effect denotes the physical occupation of interparticle space by sufficiently small particles, which improves particle-size grading and reduces initial void volume; it is distinct from later pore refinement caused by hydration or pozzolanic reaction [54].

Nano-SiO_2_ is the most extensively studied zero-dimensional modifier because its high specific surface area and pozzolanic activity strongly affect strength development. Early studies compared ANNs with conventional statistics. Using 264 experimental records for high-performance concrete containing nano-SiO_2_ and copper slag, Chithra et al. developed multiple regression analysis (MRA) and ANN models; the ANN trained with the Levenberg–Marquardt algorithm substantially outperformed MRA [55]. As datasets expanded, model comparisons became more systematic. Farshbaf Maherian et al. compiled 1143 records for concretes containing nano-SiO_2_ and supplementary cementitious materials and compared ANNs, RFs, and SVMs. RF (R^2^ = 0.93) and ANN (R^2^ = 0.92) performed best, demonstrating the potential of ML-assisted mixture design [6]. Albaijan et al. evaluated 11 algorithms for uniaxial compressive strength while considering physical consistency. Only support vector regression (SVR) and nu-support vector regression (NuSVR) exhibited physically plausible responses to parameter changes, emphasizing that predictive accuracy must be considered together with physical validity [56].

**Figure 6:**
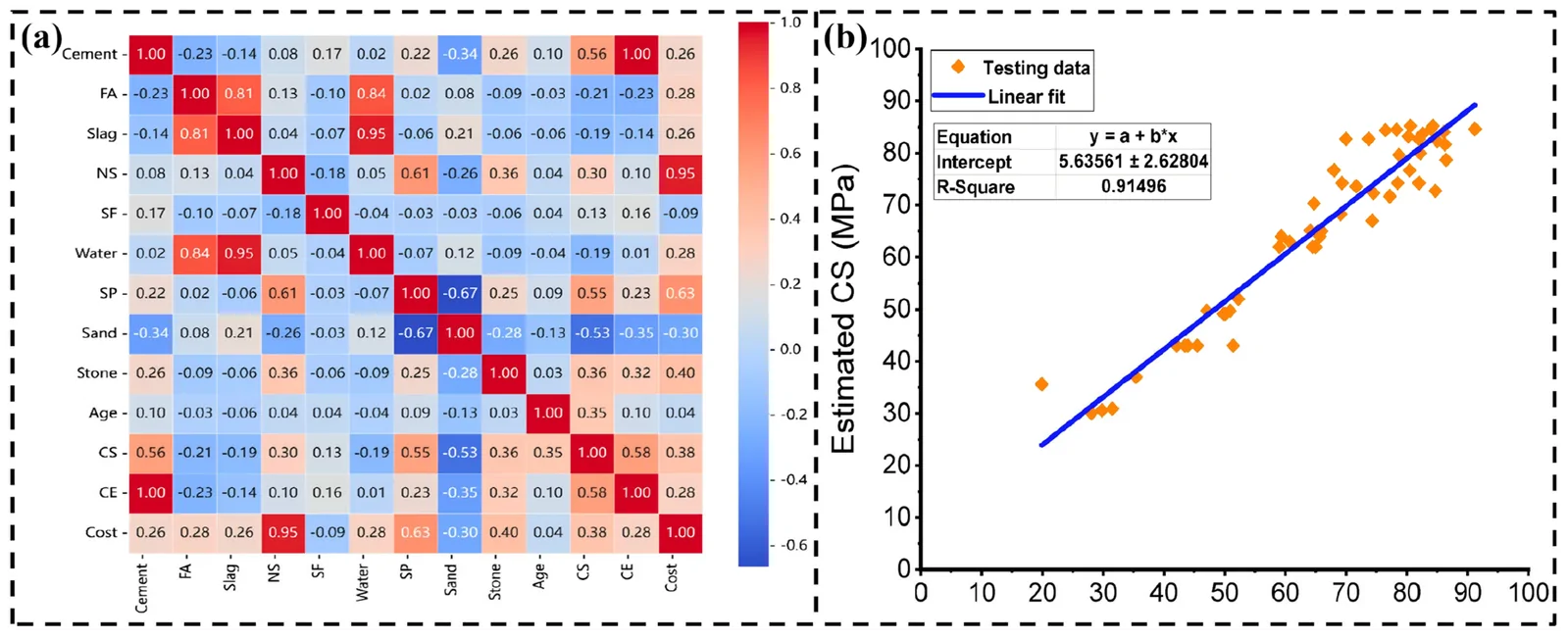
ML analysis of zero-dimensional nanoparticle-modified cementitious materials: (**a**) parameter-correlation heatmap. Reproduced with permission [41]. Copyright 2025, MDPI; and (**b**) AdaBoost prediction fit. Reproduced with permission [42]. Copyright 2022, MDPI. In panel (**b**), the asterisk (*) denotes multiplication in the linear-fit equation.

Applications have progressively expanded from strength to durability. Arasteh-Khoshbin et al. used a small dataset for nano-SiO_2_- and rice-husk-ash-containing materials and hyperparameter-optimized RF and ANN models to jointly predict water-penetration depth, chloride resistance, and compressive strength. Test-set R^2^ values ranged from 0.858 to 0.990, suggesting that appropriate optimization can reduce experimental demand [40]. Nasr et al. used an ANN to predict both mechanical properties and durability indicators, including electrical resistivity and rapid chloride penetration, for self-compacting mortar containing natural zeolite and nano-SiO_2_ [57]. For mixture optimization, Gu et al. developed an XGBoost model (R^2^ = 0.99) for nano-SiO_2_ concrete and coupled it with the non-dominated sorting genetic algorithm II (NSGA-II) to optimize strength, cost, and carbon emissions. Feature analysis showed correlations of 0.82 and 0.85 between nano-SiO_2_ dosage and strength and cost, respectively (Figure 6a), providing an operational framework for multi-objective mixture design [41].

Combining nano-SiO_2_ with fibers is a common route to improved toughness, but the larger number of components and interactions increases the need for ML. Liu et al. coupled a three-layer backpropagation (BP) neural network with a GA to predict strength and optimize mixtures of polyvinyl-alcohol (PVA)-fiber-reinforced cementitious composites containing nano-SiO_2_; orthogonal experiments were used to optimize network parameters and complete a prediction-to-optimization loop [58]. Zhang et al. constructed a hybrid radial-basis-function and backpropagation (RBF-BP) network to simultaneously predict compressive strength and chloride-penetration charge for the geopolymer mortar containing PVA fibers and nano-SiO_2_. The hybrid network converged faster and predicted more accurately than either individual model [59]. Anjum et al. compared four ensemble methods for fiber-reinforced nano-SiO_2_ concrete. AdaBoost performed best (R^2^ = 0.92; Figure 6b), and SHAP indicated a negative influence of the coarse-to-fine aggregate ratio and a positive influence of curing age [42]. Shokrnia et al. coupled an adaptive neuro-fuzzy inference system (ANFIS) and an extreme learning machine (ELM) with the gray wolf optimizer (GWO), PSO, and water cycle algorithm (WCA). ELM-GWO gave the best strength prediction (R^2^ = 0.951), illustrating the ability of metaheuristics to improve training and generalization [60].

Machine learning is also valuable in alternative binders and under extreme conditions. Khan et al. trained decision-tree, RF, XGBoost, and AdaBoost models using 420 strength records and 240 acid-resistance records for recycled-aggregate concrete (RAC) incorporating chemically activated fly ash and nano-SiO_2_. XGBoost predicted strength with R^2^ = 0.995, while RF predicted acid resistance with R^2^ = 0.909 [43]. Sobuz et al. produced high-strength concrete using metakaolin and modified recycled coarse aggregate (MRCA). XGBoost achieved R^2^ = 0.924, and SHAP and PDP analyses quantified individual factor contributions [61]. The same group compiled 380 mixture records for nano-SiO_2_ concrete exposed to elevated temperatures and compared eight models. Gradient boosting performed best (R^2^ = 0.980); SHAP identified temperature and water content as the dominant variables, and PDP analysis suggested a nano-SiO_2_ content below approximately 15 kg m^−3^ [53]. Kooshkaki et al. proposed a multi-output artificial neural network (MOANN) that included porosity as an input and simultaneously predicted the compressive and flexural strengths of silica-fume/nano-SiO_2_ mortars, demonstrating the value of explicit microstructural descriptors [62]. Multiscale sulfate-attack modeling likewise identifies phase assemblage and microstructural evolution as key strength-related descriptors [63].

Although nanoclay, nano-Al_2_O_3_, nano-ZrO_2_, and nano-CaCO_3_ have smaller evidence bases than nano-SiO_2_, distinct ML applications have emerged. Mohammed et al. applied nonlinear regression and ANNs to rheological parameters and compressive strength of nanoclay-modified cement paste and identified nanoclay dosage as the most influential variable [64]. Dahish and Alturki compared XGBoost and RF models for strength degradation of concrete containing nanoclay and nano-SiO_2_ at 25–800 °C and supplemented them with SHAP, individual conditional expectation, and PDP analyses. The study proposed optimum ranges of 2–3% nano-SiO_2_ and 5–6% nanoclay and identified temperature as the strongest negative factor [65]. Li et al. combined ANNs with SHAP to predict the strength and mass loss of shotcrete containing nano-Al_2_O_3_ sol and CaCO_3_ whiskers for high-geothermal tunnel applications and identified a favorable synergistic combination [66]. Issa et al. investigated the combined reinforcement of ZrO_2_ and multi-walled carbon nanotubes (MWCNTs) in a high-strength cementitious composite containing 15% silica fume. XGBoost achieved a test-set R^2^ of 0.990 for 301 records and suggested an optimum combination of 2.0% ZrO_2_ and 0.075% MWCNTs [44].

The integration of ML with bio-based and green-chemistry systems is an emerging direction. For bacterial self-healing concrete containing nano-biosilica, Nagarajan et al. compared XGBoost, RF, AdaBoost, and linear regression. XGBoost (R^2^ = 0.91) captured nonlinear interactions among microbial activity, nano-silica reactions, and admixture chemistry [67]. Liu et al. grafted nano-SiO_2_ onto straw fibers and developed a GA-BP model, demonstrating the combined value of surface modification and ML for durability assessment under wet-dry cycling [68].

Four limitations characterize the current data for zero-dimensional nanoparticles. First, research is concentrated on nano-SiO_2_, while ML studies of nano-MgO, nano-Fe_2_O_3_, and several other particles remain scarce. Second, most datasets are generated within a single experimental program, limiting cross-study generalization. Third, many models remain black boxes and do not systematically incorporate physical mechanisms, microstructural descriptors such as porosity, or explainability. Fourth, prediction–optimization loops, including those based on NSGA-II, remain at an early stage and require validation under engineering conditions.

A principal advantage of zero-dimensional particle datasets is that mixture variables are readily structured for nonlinear tree-based and ensemble models. Their principal weakness is that particle-size distribution, specific surface area, surface chemistry, and actual dispersion state are frequently reduced to a material label or single dosage. Models may therefore treat nanoparticles from different sources as equivalent inputs. Future databases should report particle properties, dispersion energy, admixture system, and pore-structure indicators and should use source-wise external validation to determine whether a model generalizes beyond a particular experimental dataset. Feature importance, Shapley additive explanations (SHAP), and partial-dependence trends identify statistical associations rather than causal mechanisms. Claims concerning packing, nucleation, pozzolanic reaction, or interfacial transition-zone densification therefore require corroboration by independent calorimetry, phase analysis, microscopy, or transport measurements.

Nano-modification should also be evaluated against conventional mixture-design strategies. Supplementary cementitious materials and conventional fibers can provide bulk binder replacement, crack control, and established field handling, whereas nanomaterials can act at a lower dosage and more directly regulate nucleation, interfaces, transport paths, or functional response. The latter advantages are offset by dispersion sensitivity, characterization demands, and cost. Recent mortar research using ternary binders and recycled turbine-blade fibers demonstrates that strength, workability, durability, and resource substitution can be adjusted without nanoscale modifiers [69]. Similarly, ML-optimized low-carbon composite flocculants for engineering slurry illustrate how hybrid algorithms can search multicomponent formulations under environmental constraints [70]. Recent studies on HS-ECC strengthening, strain-hardening alkali-activated basalt/slag composites, and LC3-HS-ECC further demonstrate that structural recovery, strength–ductility synergy, and bond performance can also be improved through matrix and interface design [71,72,73]. These studies are useful comparators, but they do not by themselves establish superiority or transferability to nano-modified matrices. Fair comparisons require equivalent binder content, curing and exposure conditions, functional unit, and uncertainty reporting. In comparative terms, mixture proportions, nanoparticle dosage, age, and exposure condition are the dominant inputs; strength is the main target; tree ensembles and artificial neural networks are the best-supported model classes; most validation remains internal; and missing particle-size, specific-surface-area, surface-chemistry, and dispersion descriptors limit generalization. Engineering maturity is consequently moderate for nano-SiO2 and lower for less-studied zero-dimensional particles.

### 3.2. One-Dimensional Nanomaterials

One-dimensional nanomaterials, including CNTs, CeNFs, CNCs, and CNFs, have attracted considerable interest for nanoscale reinforcement and toughening because of their high aspect ratios, large specific surface areas, and favorable intrinsic mechanical properties. They can bridge microcracks, fill pores, and form three-dimensional networks among hydration products. Their effects are not linearly proportional to dosage, however, and depend strongly on dispersion, geometry, orientation, and interfacial bonding [34,35]. Achieving uniform dispersion while retaining predictable reinforcement is therefore a central challenge. ML provides a means of representing the resulting composition–processing–property relationships [36,45].

Numerous experiments have shown that CNTs can improve compressive strength, flexural strength, and toughness. Manan et al. used 295 records to develop a gene expression programming (GEP) model for the compressive strength of CNT-reinforced cementitious composites. The water-to-cement ratio and CNT geometry were the most influential variables, and the best model achieved R^2^ = 0.92 on validation data [34]. Wang et al. conducted impact-compression tests on composites containing different MWCNTs and found that short, thick MWCNTs at 0.25% increased impact toughness by as much as 100.8%, primarily through crack bridging [35]. Cui et al. compared 12 MWCNTs with different dimensions, functional groups, and structures. Short, thick MWCNTs designated SM5 increased compressive strength by 47.1% at 0.8% and flexural strength by 55.2% at 0.1%, whereas hydroxyl-functionalized SMH1 increased flexural strength by 76% at 0.5% [74]. Thus, reinforcement efficiency depends jointly on the dosage and intrinsic CNT characteristics. Zhu et al. proposed a stress–strain constitutive model incorporating CNT and steel-fiber contents [75], while Wang et al. combined hydration kinetics and micromechanics in a multiscale simulation of the elastic modulus and revealed the influence of CNT orientation [76]. Eisa et al. further reported that 0.5% CNTs reduced rut depth in asphalt concrete by 45%, demonstrating reinforcement across different binder systems [77].

**Figure 7:**
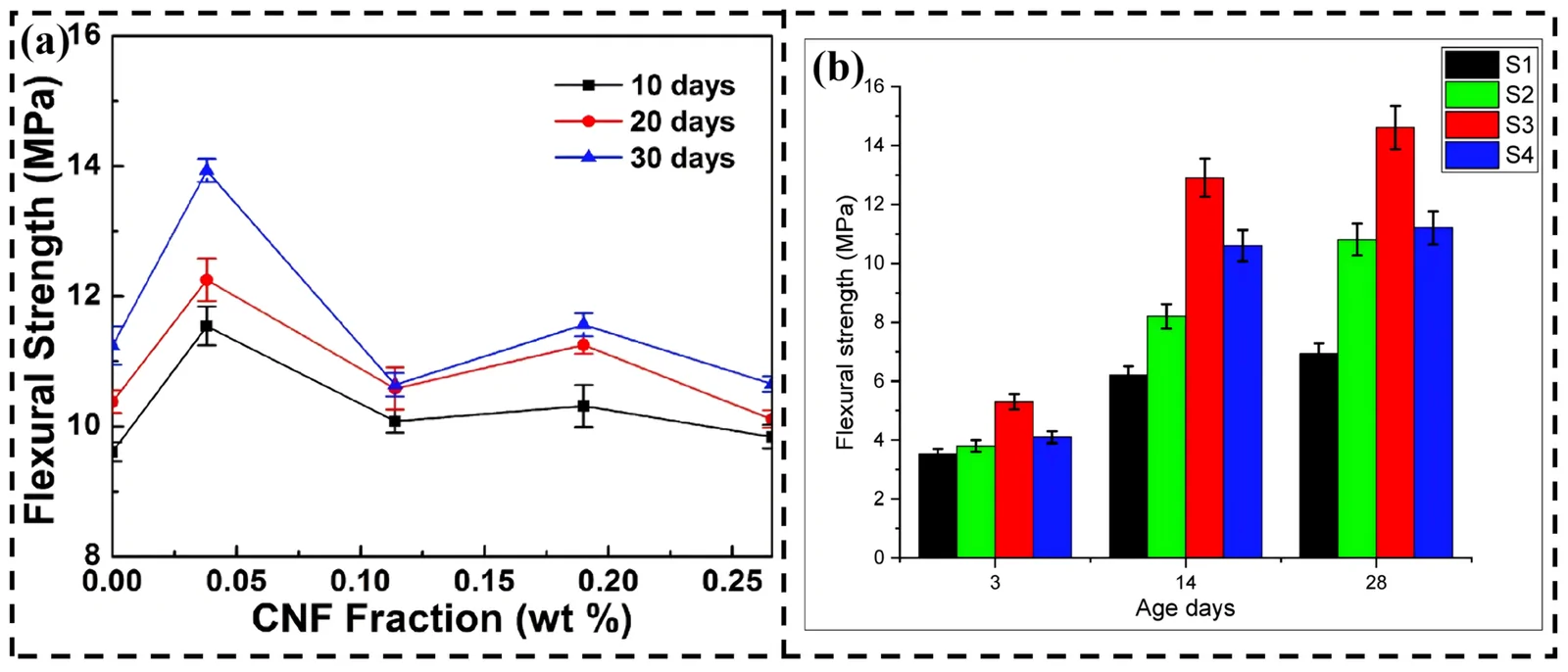
Reinforcement and optimization of cementitious materials with one-dimensional nanomaterials: (**a**) effect of CeNF dosage on flexural strength. Reproduced with permission [14]. Copyright 2016, Scientific Reports; and (**b**) flexural strength of hybrid CNT/GNP systems. Reproduced with permission [78]. Copyright 2020, Materials.

Cellulose nanocrystals and CeNFs provide renewable, high-strength reinforcement. Sun et al. investigated CeNFs in oil-well cement paste and found that a dosage of 0.04% increased flexural strength by 20.7%; the rheological response was accurately described using the Vom Berg model (Figure 7a) [14]. Taheri et al. used microfibrillated cellulose to produce ultralight cementitious composites with densities of 450–800 kg m^−3^. Although compressive strength decreased with increasing porosity, fiber bridging limited the loss of flexural strength and partially suppressed drying shrinkage [15]. Molecular dynamics (MD) simulations by Zhang et al. indicated an interfacial interaction energy of −1.09 J m^−2^ between CeNFs and C-S-H, compared with −0.046 J m^−2^ between CNTs and C-S-H. The stronger interaction was attributed to hydrogen bonding between surface hydroxyl groups and C-S-H, which increased bridging and pull-out resistance [16].

Carbon nanofibers have diameters intermediate between CNTs and conventional carbon fibers and can provide both nanofilling and microfiber bridging. Chen et al. compared carbon fibers (CFs) and CNFs under static and dynamic loading. CNFs more effectively increased static strength through filling, whereas CFs dissipated more energy under dynamic loading through crack arrest [17]. In ultra-high-performance concrete (UHPC), Meng et al. found that 0.3% CNFs reduced total porosity by approximately 35%, while plastic viscosity varied non-monotonically with dosage [18]. Farooq et al. reported that a hybrid containing 0.04% CNTs and 0.04% graphene nanoplatelets (GNPs) improved CNT dispersion and increased flexural strength by 110% through combined crack bridging and deflection (Figure 7b) [78]. MacLeod et al. used a predispersed liquid CNT admixture and extended mixing to 15 min, increasing 28-day concrete strength by 21–37% and demonstrating the importance of processing variables [79].

One-dimensional nanomaterials can also improve durability. Konsta-Gdoutos et al. studied corrosion resistance of CNT- and CNF-modified mortar electrochemically. Mortar containing 0.1% CNTs remained in the passive region after 150 days in 3.5% sodium chloride (NaCl), which was attributed to pore refinement and denser C-S-H. At 0.5%, however, poorly dispersed CNT agglomerates may have promoted microgalvanic corrosion and reduced durability [80]. Hawreen et al. found that 0.05–0.1% CNTs reduced early-age shrinkage by 62% and increased fracture energy by 65% [81]. Tragazikis et al. used acoustic emission (AE) for fracture monitoring of CNT-modified mortar. At 0.4% MWCNTs, both AE energy and the rise-angle-to-amplitude (RA) value reached maxima, indicating a change in the fracture mode from shear to tension and providing a nondestructive means of assessing nano-modification [82].

Dispersion remains a primary barrier to engineering use of CNTs. Kim et al. reported that polycarboxylate superplasticizers overcome van der Waals attraction through an adsorption layer of approximately 100 Å, while the ball-bearing effect of silica fume further promotes uniform distribution in cement paste [25]. Jung et al. introduced oxygen-containing carboxyl groups by ozone treatment at only 0.1 ppm. Electrical-double-layer repulsion stabilized CNTs in water and increased the 28-day compressive strength of UHPC by 9.8% [26]. Cui et al. presaturated sand surfaces with CNTs, strengthening the ITZ and increasing the 28-day compressive strength of low-carbon calcium sulfoaluminate concrete by 27.8%. MD simulation suggested that adsorption of Ca^2+^ by CNTs promoted nucleation of ettringite (AFt) and aluminum hydroxide (AH_3_) at the interface [83]. de Souza et al. synthesized MWCNTs in situ on clinker, avoiding a separate dispersion step; at 0.15%, compressive and tensile strengths of the paste increased by 27% and 43%, respectively [84]. Li et al. developed a viscosity model that accounts for CNT adsorption and entanglement and quantified the effects of aspect ratio and functional groups, supporting mixture design for three-dimensional printing and related construction processes [85].

Machine learning offers an alternative to trial-and-error exploration of multicomponent, multiscale CNT systems. Yan et al. assembled 429 records covering 11 factors, including CNT morphology, dispersion process, and curing age. A PSO-optimized XGBoost model achieved R^2^ = 0.91 on the test set, and the SHAP analysis suggested a CNT dosage of 0.1–0.3% and an inner diameter of at least 7.13 nm [45]. Çiftçioğlu et al. coupled GWO with XGBoost to predict the mechanical properties of UHPC containing CNTs and other nanomaterials and obtained R^2^ values up to 0.984 [36]. Salih et al. combined ML with NSGA-II to optimize cost, strength, and environmental impact and generated 115 Pareto-optimal mixtures [86]. Manan et al. compared GEP, ANN, and RSM models and found that GEP provided the best generalization for compressive strength [34]. Mirindi et al. used decision trees and hierarchical clustering to analyze mechanical data for concrete containing several nanoparticles, identifying nanoparticle concentration as the principal factor and separating clusters associated with different reinforcement mechanisms [87]. Collectively, these studies show that ML can combine prediction, feature interpretation, and experimental search-space reduction.

In summary, CNTs, CNCs, CeNFs, and CNFs reinforce and toughen cementitious materials through bridging, filling, nucleation, and multiscale interactions. Their effectiveness nevertheless depends strongly on dispersion, geometry, interface, and mixture design. The integration of ML with MD simulation provides a route from empirical testing toward mechanism-aware design. Progress toward practical use will require standardized multidimensional databases and interpretable hybrid models.

From a modeling perspective, the key distinction between one-dimensional nanomaterials and zero-dimensional particles is that reinforcement efficiency is jointly governed by dosage, geometry, interface, and orientation. Many current models include only CNT or CNF mass fraction and omit length, diameter, aspect ratio, functionalization, ultrasonication energy, and dispersion stability. Such simplification restricts identification of bridging thresholds, percolation networks, and agglomeration. Integrating microscopy, Raman or infrared spectra, and electrical responses with formulation data through multimodal learning and multiscale simulation could establish a continuous predictive chain from dispersion state to crack evolution and macroscopic performance. For electrical and sensing targets, models should additionally encode percolation onset, network connectivity, tunneling distance, contact resistance, and strain-dependent network disruption. Dispersion quality may be included directly through microscopy, spectroscopy, zeta-potential, or stability measurements, or represented as a latent microstructural variable in multimodal models. Overall, typical datasets remain below several hundred records, external validation is uncommon, and incomplete geometry, orientation, percolation, and dispersion descriptors place current engineering maturity at a low-to-moderate level.

### 3.3. Two-Dimensional Layered Nanomaterials

Unlike zero-dimensional particles and one-dimensional fibers, 2D layered nanomaterials, including GO, h-BN, MoS_2_, and LDHs, consist of atomically thin sheets with large specific surface areas and pronounced geometric anisotropy. Their reinforcement mechanisms include sheet bridging that suppresses microcrack growth, barrier effects that retard penetration of aggressive species, and template-assisted nucleation of hydration products. These mechanisms complement the filling and pozzolanic effects of zero-dimensional particles and the crack-bridging action of one-dimensional materials. Graphene oxide is the most extensively studied 2D modifier because its oxygen-containing groups favor aqueous dispersion, and it was also among the first 2D materials investigated with ML. Nevertheless, its effects depend nonlinearly on sheet size, dosage, dispersion, and curing. This section reviews ML-assisted property prediction, mechanism analysis, and functional development for carbon-based sheets, h-BN, MoS_2_, and LDHs, with particular emphasis on GO.

**Figure 8:**
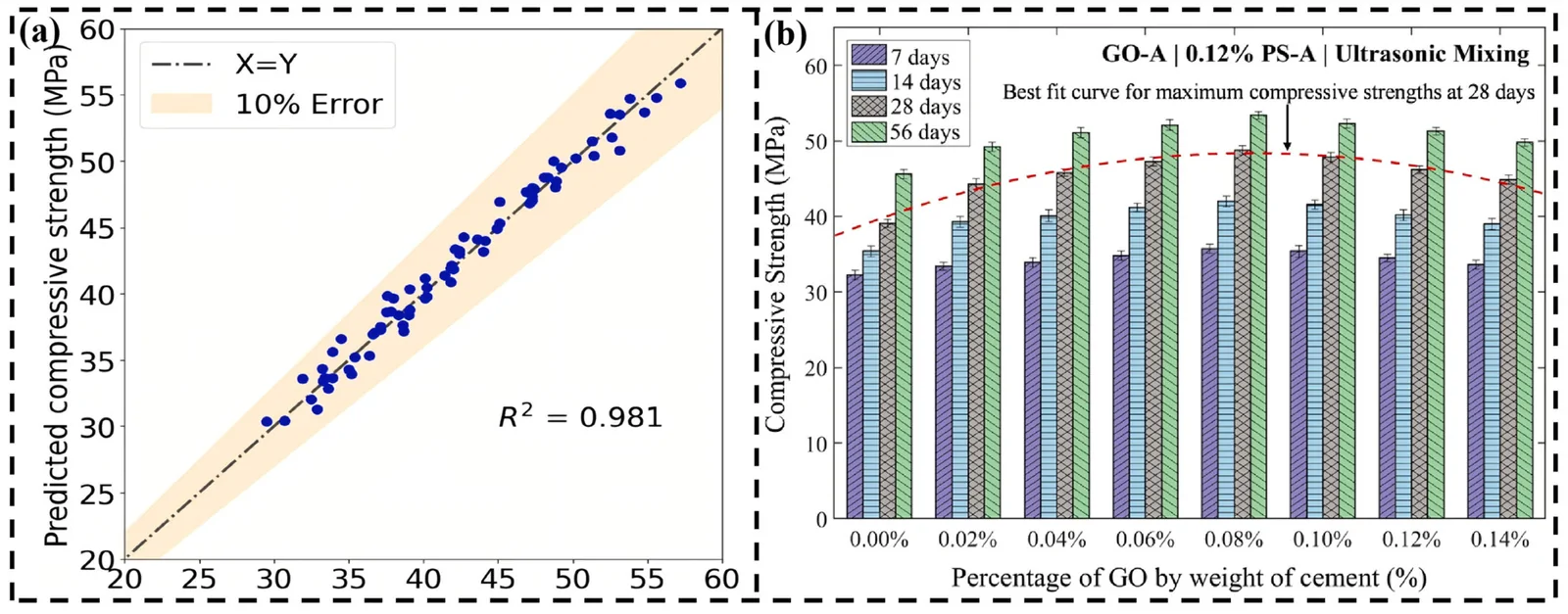
Multiscale ML workflows for two-dimensional nanomaterials in cementitious systems: (**a**) XGBoost predictions of measured compressive strength for GO concrete (R^2^ = 0.981). Reproduced with permission [46]. Copyright 2024, Construction and Building Materials; and (**b**) compressive strength of concrete containing different dosages of GO-A ultrasonically dispersed with superplasticizer A (PS-A). Reproduced with permission [90]. Copyright 2024, Construction and Building Materials.

For GO concrete, ML has progressed from individual prediction models to workflows combining explainability and hybrid optimization. Meddage et al. constructed a compressive-strength dataset containing GO type and dosage, superplasticizer type and dosage, dispersion method, and curing age. Among multiple linear regression (MLR), k-nearest neighbors (KNN), RF, and XGBoost, XGBoost performed best (R^2^ = 0.981; Figure 8a). The explainable analysis identified optimum GO and superplasticizer dosages consistent with experiments [46]. Rout et al. coupled RF with several metaheuristics; starfish-optimization-algorithm-optimized RF (SOA-RF) achieved a test-set R^2^ of 0.93 for 367 samples. SHAP identified cement content, curing age, and GO dosage as the dominant variables, and the authors developed a Streamlit interface for practical use [47]. For high-performance concrete containing GO and silica fume, Rao et al. compared eight supervised algorithms using 750 specimens. RF performed best, with a root mean square error (RMSE) of 2.47 MPa and R^2^ = 0.776, and consistently identified silica fume, GO, and cement as the principal factors [48]. Graphene nanoplatelets have also been modeled. Fawad et al. used CatBoost for GNP-reinforced cementitious composites and reported a correlation coefficient of 0.9999; SHAP ranked GNP thickness above diameter, curing age, and water-to-cement ratio [88]. Alyousef et al. compared ANN, GEP, and ANFIS models for strength and electrical resistivity and found that the GEP performed best; curing age dominated strength, whereas GNP dosage dominated resistivity [89]. Collectively, these studies reveal nonlinear dosage–dispersion–strength relationships and a recurring optimum beyond which agglomeration degrades performance.

Dosage alone does not determine the benefits of 2D nanomaterials; dispersion and processing can be equally or more influential. Fonseka et al. compared two commercial GO products, two superplasticizers, and mechanical, high-speed, and ultrasonic dispersion. Optimum dosages were 0.08% for GO-A and 0.12% for GO-B (Figure 8b), and ultrasonication combined with a compatible superplasticizer increased 28-day compressive, splitting-tensile, and flexural strengths and the elastic modulus by 33%, 24%, 25%, and 30%, respectively [90]. These results demonstrate the need to encode GO properties and dispersion protocols explicitly. At the characterization level, manually interpreted infrared spectra often fail to distinguish GO samples with strongly overlapping bands. Filatov et al. combined ML with attenuated total reflectance Fourier-transform infrared spectroscopy (ATR-FTIR) to automate GO identification and functional-group assignment, providing a high-throughput route for batch discrimination, quality traceability, and improved training data [91].

Machine learning can also connect macroscopic performance with pore-size distribution and multifunctional behavior. Yu et al. combined metal impregnation, backscattered-electron imaging, and DL-based segmentation to quantify pores in GO-modified UHPC with 95% accuracy [92]. GO reduced matrix porosity by 12.6–32.6% and ITZ porosity by 13.3–26.7%; the best-performing modified GO formulation produced a 27.2% reduction. Compressive strength correlated with total porosity (goodness of fit = 0.85), and pores of 10–15 μm exerted the strongest influence (goodness of fit = 0.915). For self-sensing applications, Alsaif et al. used extremely randomized trees to predict the electrical resistivity of nano-graphite cementitious composites. SHAP identified the test method, ultrasonication time, and graphite dosage as the most important variables [93]. Ramana et al. combined ANNs and RSM to predict the mechanical properties of fly-ash/GO concrete exposed to 200–800 °C, extending ML applications to thermal durability and fire resistance [37].

Hexagonal boron nitride has a graphene-like honeycomb lattice but is an electrically insulating wide-bandgap material. Its use in cementitious materials is at an early stage, and current ML studies primarily address production and nanoscale mechanics. Varughese et al. modeled the yield of h-BN nanosheets produced by ultrasonication-assisted liquid exfoliation. An Adam-optimized deep neural network (DNN) achieved R^2^ = 0.984 and enabled rapid optimization of sonication power, duration, and solvent composition [94]. Mousavi et al. integrated MD, a convolutional neural network (CNN), and transfer learning to predict the elastic modulus and ultimate tensile strength of defective h-BN monolayers, with mean absolute errors (MAEs) of 13.21 and 3.81 GPa, respectively. An inverse-design module generated defect configurations with target mechanical properties [95]. This workflow is methodologically relevant to future h-BN-reinforced construction materials, but direct validation in such matrices remains necessary.

Machine-learning applications to MoS_2_ and LDHs in construction materials remain sparse. Most ML studies of MoS_2_ concern electronic or mechanical devices. Ding et al., for example, used explainable ML and DL to decode tribovoltaic signals from a self-sensing bearing and identified rotational speed with more than 96% accuracy [96]. The surface-as-sensor concept may inform structural-health-monitoring materials but has not yet been demonstrated in a construction matrix. For LDHs, the lack of systematic datasets has likewise prevented mature predictive studies. More broadly, 2D-material research is expanding from mechanical prediction toward integrated structural and functional design. Sun et al. trained a DL model on 100,000 records for carbon-dioxide adsorption by GO-based materials; an ANN achieved R^2^ = 0.988 on the test set and identified adsorption pressure and micropore volume as the principal descriptors [49]. Vairagade et al. combined nano-SiO_2_, GO, and CNTs in a ternary high-performance concrete and used RF, gradient boosting, and a DNN to predict strength and chloride-penetration indicators. GA and PSO optimization increased strength by 15% and durability by 30% while reducing cement content [97].

Overall, ML applications to 2D nanomaterials have expanded from macroscopic strength prediction to pore segmentation, resistivity prediction, and inverse design, but direct evidence in construction materials remains concentrated on GO and GNPs. Reported ML studies of h-BN, MoS_2_, and LDHs mostly address nanosheet production, monolayer mechanics, or unrelated devices and lack matrix-specific training data and independent validation. Future studies should establish standardized datasets containing sheet size, layer number, defect density, surface charge, dispersion state, and environmental stability. Model-derived influential variables and optimum ranges should then guide formulation, followed by sequential validation through dispersion characterization, microstructural analysis, and macroscopic testing. This would create an iterative workflow of data modeling, parameter screening, experimental validation, and model updating. The evidence levels are not equivalent: GO has the most direct construction-matrix support; graphene nanoplatelets have a smaller but construction-relevant evidence base; h-BN and MoS_2_ are presently supported mainly by transferable production, nanoscale-mechanics, or sensing workflows; and ML data for LDHs in construction matrices remain insufficient. This hierarchy should be considered when interpreting model accuracy and proposed optimization routes.

### 3.4. Functional Nanomaterials

Functional nanomaterials, including nano-TiO_2_, nano-ZnO, and nano-PCMs, can impart photocatalytic self-cleaning, resistance to ultraviolet aging, thermal-energy storage, temperature regulation, and condition-sensing functions to construction materials. Unlike nanomaterials used primarily for filling or reinforcement, these systems usually involve competing targets. Photocatalytic activity must be balanced against surface durability; anti-aging performance must be assessed across high- and low-temperature mechanical responses; and thermal-regulation capacity may conflict with load-bearing performance or cyclic stability. Crystal phase, particle size, surface exposure, matrix compatibility, and service environment further affect functional response. Consequently, ML should extend beyond fitting a single functional indicator to support nanomaterial screening, processing optimization, multi-property prediction, and long-term service assessment [22,98,99].

**Figure 9:**
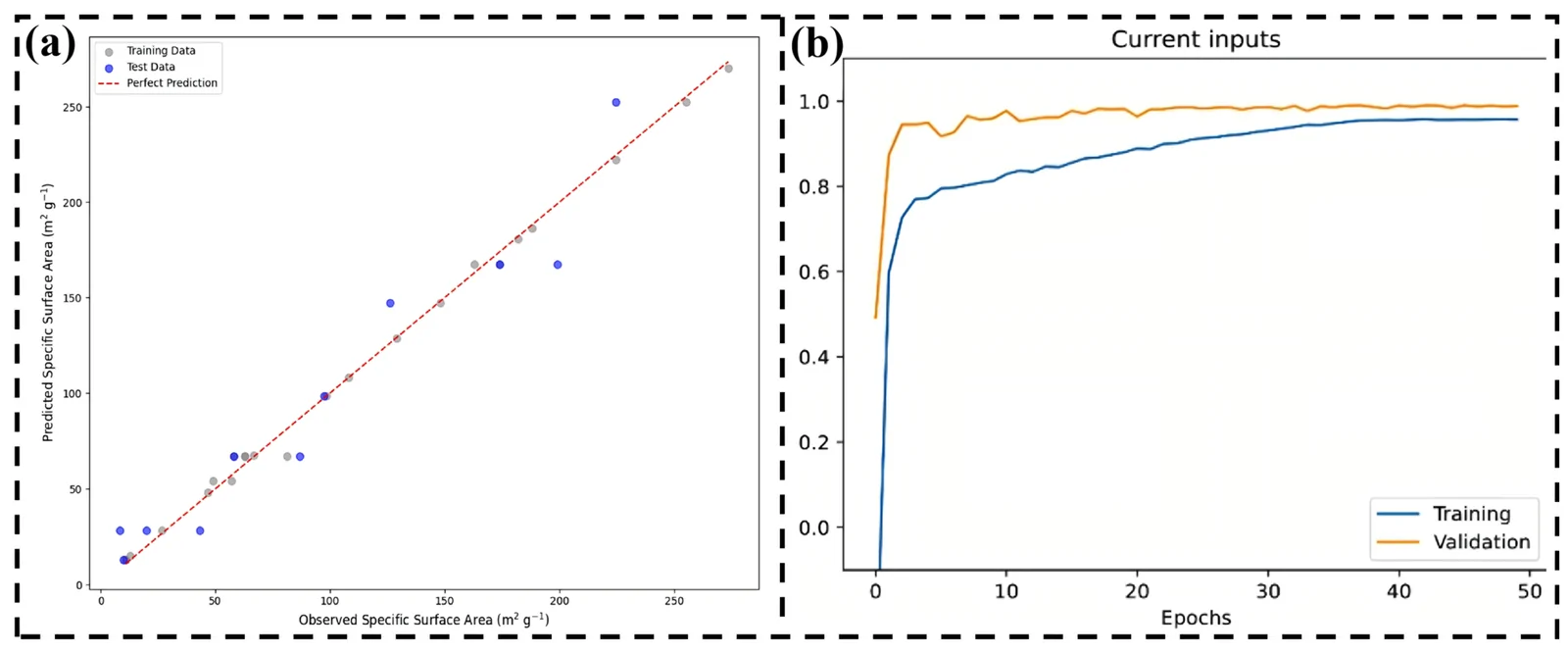
ML-assisted prediction and optimization of functional nanomaterials: (**a**) prediction of the specific surface area of Nb_2_O_5_ nanoparticles. Reproduced with permission [100]. Copyright 2026, Surfaces and Interfaces; and (**b**) training and validation performance of the CNN model. Reproduced with permission [107]. Copyright 2024, Sensors and Actuators A: Physical.

In photocatalytic materials and functional coatings, ML supports three principal tasks: prediction of intrinsic properties, characterization of processing, and optimization of service performance. da Silva et al. used synthesis route, metallic precursor, and heat-treatment temperature as inputs and compared RF, XGBoost, and gradient-boosting regression for predicting the specific surface area and bandgap of Nb_2_O_5_ nanoparticles. Gradient boosting achieved a test-set R^2^ above 0.90, demonstrating data-driven screening of synthesis conditions (Figure 9a) [100]. Heger et al. combined neural networks with grazing-incidence small-angle X-ray scattering (GISAXS) to infer the domain-size distribution of biopolymer-templated porous TiO_2_ coatings from two-dimensional scattering patterns during spray deposition [99]. Mehta et al. used gradient boosting, light gradient-boosting machine (LightGBM), XGBoost, and CatBoost to predict wear and friction of TiO_2_/Al/TiO_2_ multilayer coatings [101]. These studies show that coating models should jointly record intrinsic nanomaterial properties, coating structure, substrate characteristics, and deposition parameters; the dosage alone cannot adequately explain performance.

For nano-modified asphalt, ML has primarily addressed aging-state identification, full-temperature rheology, and performance-cost optimization. Zhao et al. screened chemical and rheological indicators for nano-ZnO/SBR-modified asphalt and developed an aging equation based on asphaltene content. Nano-ZnO retarded conversion of light fractions and polymer degradation [22]. Ren et al. combined Fourier-transform infrared spectroscopy (FTIR), atomic force microscopy (AFM) nanomechanics, and rheological parameters in an explainable ML framework. XGBoost and RF achieved R^2^ values above 0.9, and SHAP revealed changes in the dominant mechanism across temperature ranges [50]. Guo et al. coupled a performance model for nano-TiO_2_/CaCO_3_- and basalt-fiber-modified asphalt with life-cycle assessment (LCA) and an improved NSGA-II to jointly optimize performance, cost, and environmental indicators [98]. Hosseinian et al. used a multilayer perceptron (MLP) to predict fracture toughness and fracture energy of nano-CaCO_3_-modified warm-mix rubber asphalt and used NSGA-II to recommend dosages under different loading modes [102]. Asphalt models should therefore include temperature, loading frequency, aging history, and compositional evolution in addition to nanomaterial descriptors.

In thermal-management materials, ML can predict heat transfer, guide coating design, and support multi-objective optimization of phase-change systems. Kumar et al. compared SVR, least-squares support vector regression (LSSVR), Gaussian process regression (GPR), ELM, and ANN models for the pool-boiling heat-transfer coefficient of CNT/GO nanocoatings; GPR provided the highest accuracy [51]. Wang et al. developed a bidirectional DNN for transparent SiO_2_@Ag/poly(methyl methacrylate) (PMMA) coatings, enabling forward prediction of color and photovoltaic response and inverse design of structural parameters [103]. Liu et al. used RSM to relate structural parameters of hydrophobic nano-SiO_2_/CNT coatings to ice-adhesion strength, supporting anti-icing design for built surfaces [104]. Heat-transfer studies of ZnO and SiO_2_ nanoarrays further show that DNNs, long short-term memory (LSTM) networks, and SHAP can identify influential variables such as heat flux and initial temperature [105,106]. Some of these examples originate from energy equipment or microelectronic cooling; their data structures and workflows are informative for buildings, but the trained models cannot be transferred directly to envelope materials. Nano-PCM research particularly requires datasets containing transition temperature, latent heat, shell structure, leakage rate, thermal-cycle count, matrix type, and mechanical performance.

Machine learning can additionally be combined with nanoindentation, sensor signals, and electrochemical measurements for parameter inversion and condition monitoring. Kalscheuer et al. generated nanoindentation data by finite-element simulation and used an SVR-gated recurrent unit (GRU) model to infer the elastoplastic parameters of physical-vapor-deposition (PVD) coatings from load–depth curves [108]. Wang et al. combined signals from a solid–liquid triboelectric nanogenerator (TENG) with a convolutional neural network (CNN) to classify coating corrosion and predict impedance (Figure 9b) [107]. These approaches show that images, spectra, mechanical curves, and time-series sensor data can complement conventional formulation datasets and facilitate continuous identification of functional degradation. However, major differences in testing conditions, performance metrics, and data partitioning presently favor validation within individual material systems; R^2^ values alone should not be used to rank models or materials across studies.

Functional-nanomaterial research is thus expanding from prediction of intrinsic properties toward integrated modeling of processing, structure, function, and service. Photocatalytic and protective coatings increasingly employ multimodal characterization, and nano-modified asphalt studies have begun to combine explainability with multi-objective optimization. In contrast, datasets for nano-PCMs and long-term functional retention remain limited. Progress depends less on additional isolated high-accuracy models than on matrix-appropriate descriptors, standardized illumination, temperature, aging, and cycling protocols, and validation using independent data and long-duration tests. Multi-task learning could then jointly predict functional, mechanical, and durability indicators, while BO or active learning could reduce the experimental search space. Models that predict intrinsic nanomaterial properties, such as particle yield, bandgap, or specific surface area, are therefore distinguished from models that predict composite-scale performance in cementitious, asphalt, coating, or building-envelope matrices. Only the latter provide direct construction-material design evidence; the former are treated as transferable methodological examples.

### 3.5. Carbon Dots and Emerging Hybrid Nanomaterials

Carbon dots are zero-dimensional carbon nanomaterials, typically smaller than 10 nm, with abundant surface functional groups, good aqueous dispersibility, and tunable interfacial chemistry. In construction materials, CDs have been investigated as corrosion inhibitors for reinforcing steel and may also support hydration regulation, ion adsorption, fluorescent tracing, and condition sensing. Compared with nano-SiO_2_, CNTs, and GO, published ML studies of CDs and emerging hybrid nanomaterials remain limited. Their principal methodological value lies in connecting synthesis conditions and structural descriptors with interfacial interactions and service performance, thereby improving the screening efficiency of complex nanosystems.

Corrosion protection of reinforcing steel is the representative construction-related application of CDs. They are commonly prepared by hydrothermal or solvothermal routes, and their inhibition performance depends on precursor type and amount, solvent, reaction temperature and duration, elemental composition, particle size, and surface functionality. One-factor-at-a-time experiments cannot efficiently identify interactions among these variables or infer synthesis conditions from a target inhibition efficiency. ML can represent nonlinear synthesis–property relationships, but datasets should distinguish synthesis parameters, structural descriptors, corrosive medium, and test method rather than indiscriminately merging measurements obtained under different conditions.

**Figure 10:**
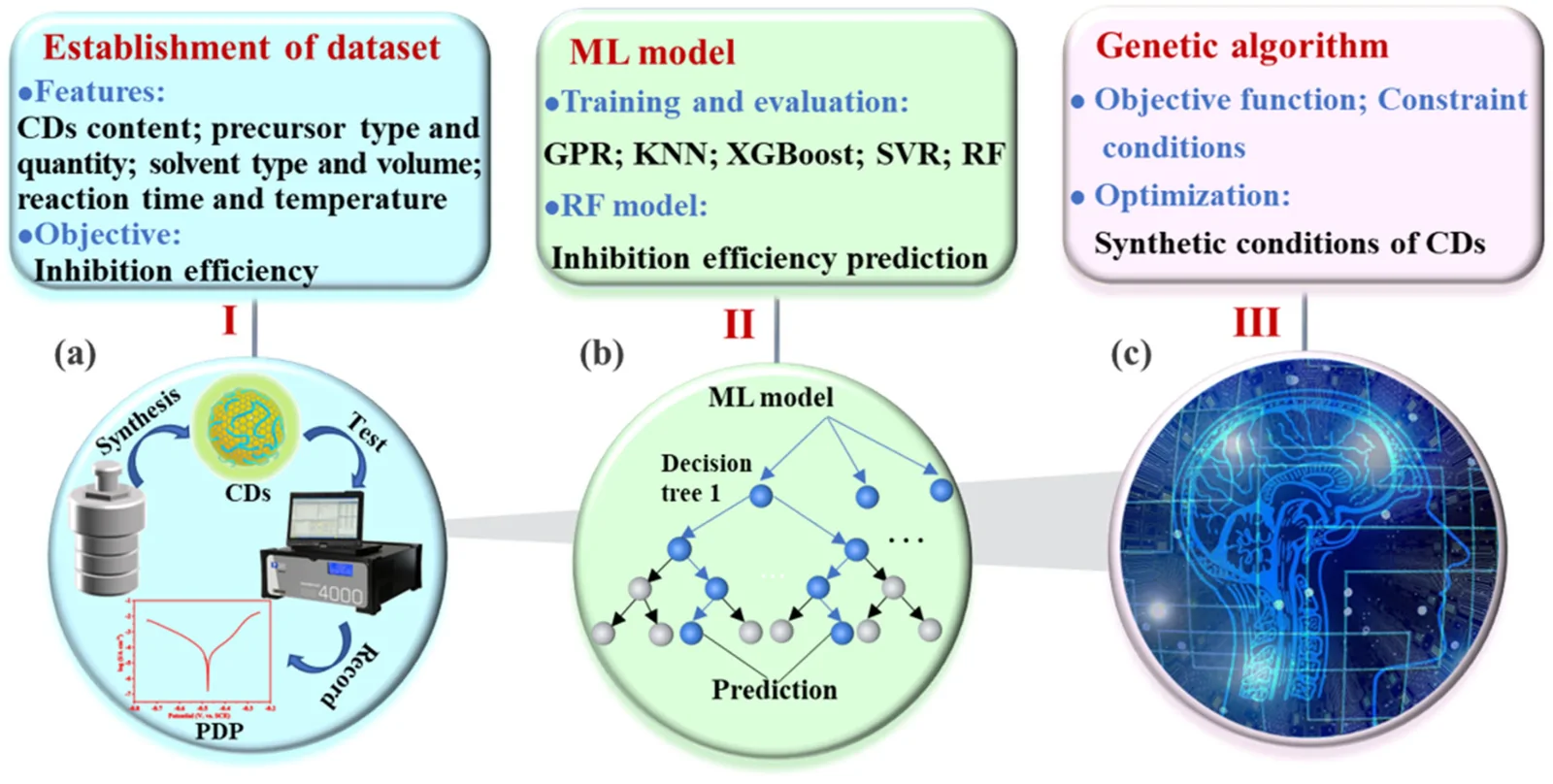
ML-assisted controllable synthesis of CD-based corrosion inhibitors: (**a**) dataset construction; (**b**) predictive modeling of inhibition efficiency; and (**c**) optimization of CD synthesis conditions. Reproduced with permission [52]. Copyright 2023, Journal of Cleaner Production.

He et al. established a workflow comprising data assembly, model training, parameter optimization, and experimental validation for CD-based corrosion inhibitors (Figure 10) [52]. A dataset of 102 literature and experimental records used CD concentration, carbon and nitrogen contents, precursor amount, solvent type and volume, reaction time, and reaction temperature as eight inputs, with potentiodynamic-polarization inhibition efficiency as the output. Gaussian process regression, KNN, XGBoost, SVR, and RF were compared using 10-fold cross-validation. RF performed best, and feature-importance analysis identified CD concentration, nitrogen content, and reaction time as the principal factors. Coupling RF with a GA optimized the hydrothermal preparation of nitrogen-doped CDs derived from o-phenylenediamine. The measured inhibition efficiency under the recommended conditions was 92.3%, compared with a prediction of 94.6%; reaction time decreased from 12.0 to 2.28 h, and water replaced ethanol as the solvent. The study demonstrates that ML can narrow the synthesis search space, although the conclusions remain based on a small dataset and a specific corrosive medium.

The current data has clear boundaries. Small sample sizes and limited sources mean that random cross-validation may overestimate generalization to new precursors or corrosion environments. Inputs are dominated by synthesis variables and elemental contents and insufficiently describe particle-size distribution, zeta potential, surface functionality, adsorption configuration, and interactions with the construction-material matrix. Optimization has also focused on a single inhibition-efficiency target without systematically considering hydration compatibility, workability, mechanical performance, ion transport, or long-term stability. Future studies should therefore use grouped validation by literature source or experimental batch and independently test model-recommended syntheses and corrosion performance.

Multicomponent nano-hybridization is another emerging direction. Combining zero-dimensional particles with CNTs or GO may integrate filling, bridging, and barrier effects, while combinations of functional nanoparticles with fibers, PCM microcapsules, or protective coatings may coordinate mechanical, durability, and functional objectives. Increasing component count, however, enlarges the parameter space and introduces synergy, competition, and agglomeration. Tree-based or ensemble models can identify nonlinear interactions; SHAP can screen influential combinations; and NSGA-II, BO, or active learning can generate multi-objective candidates. A material name should be decomposed into comparable descriptors, including size, morphology, surface chemistry, dispersion process, and matrix compatibility.

Machine-learning studies of CDs and emerging hybrid nanomaterials should presently be regarded as an emerging opportunity rather than an established construction-material platform. Existing work demonstrates the feasibility of a prediction–optimization–validation loop for a small corrosion-inhibitor dataset but does not support broad extrapolation across precursors, matrices, or service environments. The next stage should prioritize larger datasets generated under harmonized test conditions, integrate spectroscopy, electrochemistry, microscopy, and molecular simulation, and evaluate corrosion inhibition, strength, durability, and functional retention through multi-task or multi-objective models. Only independent validation and closed-loop testing in actual construction-material matrices can establish engineering applicability.

### 3.6. Comparative Selection of ML Methods and Evidence Quality

No algorithm is uniformly superior. Method choice should follow data modality, sample size, task, validation structure, and the need for interpretation. Linear or response-surface models provide transparent baselines when curvature and interactions are limited. Support vector regression is useful for small nonlinear datasets but is sensitive to kernel and hyperparameter selection. Random forests and gradient-boosted trees generally perform well on heterogeneous tabular data and support feature-attribution analysis, but they do not establish causality and extrapolate poorly beyond the training domain. Artificial neural networks are flexible but become unstable or opaque when sample size is small. Deep learning is most defensible for images, spectra, and time-series signals with adequate data or transfer learning. Metaheuristic algorithms improve hyperparameter or formulation searches but can overfit a weak surrogate model; Bayesian optimization is often more sample-efficient for sequential experiments. These trade-offs are summarized in Table 4.

Cross-study model rankings are meaningful only when data partitions, target definitions, and metrics are comparable. Random splits can leak near-duplicate formulations across training and test sets; grouped splitting by publication, mixture family, or experimental batch is therefore preferred for literature-derived data. External validation uses a genuinely independent laboratory, source, or time period and should be distinguished from an internal hold-out test. Optimization should be reported as a separate stage and followed by experiments on model-recommended candidates. The principal limitations, their consequences, and the corresponding research priorities are summarized in Table 5.

## 4. Conclusions and Outlook

This review combined bibliometric mapping with a methodologically screened qualitative synthesis of ML-driven performance optimization for nano-modified construction materials. Annual output rose from four publications in 2012 to 268 in 2025, and ML produced the strongest recent keyword burst (22.57). Reported test-set R^2^ values in representative tabular studies ranged approximately from 0.858 to 0.995, but this range must not be interpreted as a league table because dataset size, leakage risk, target variance, and validation strategy differed. Quantified material outcomes included 15% strength and 30% durability improvements in an optimized ternary nanosystem and a measured corrosion-inhibition efficiency of 92.3% for an ML-optimized carbon-dot formulation. These examples demonstrate potential, while the comparative assessment shows that independent external validation and experimental verification remain uncommon.

Research maturity is closely associated with data availability. Zero-dimensional particles such as nano-SiO_2_ are supported by comparatively large structured mixture datasets, and applications have expanded from strength prediction to durability and multi-objective optimization. For CNTs and GO, attention is shifting from macroscopic properties toward dispersion, microstructure, and interfacial effects. Data for functional nanomaterials and CDs are distributed across cementitious, asphalt, coating, and thermal-management matrices, and their evaluation metrics remain inconsistent. Studies should therefore not be ranked solely by R^2^; sample size, feature completeness, data partitioning, external validation, and applicability domain must also be considered.

Future progress first requires improved data quality, reusability, and nanomaterial representation. Matrix-aware reporting standards should record the nanomaterial source, size and morphology, specific surface area, surface functionality, dispersant, dispersion energy, mixing sequence, curing or aging environment, and testing standard in addition to conventional composition and age. One-dimensional materials require length, diameter, aspect ratio, and orientation; two-dimensional materials require sheet size, layer number, defects, and surface charge; and functional materials require crystal phase, bandgap, latent heat, and cyclic stability. Cross-study datasets should preserve source and matrix labels, harmonize units, and document outliers and measurement uncertainty. Generalization should be evaluated using grouped validation by publication or experimental batch.

Second, data-driven models should be integrated with matrix-specific mechanisms and evaluated more rigorously. Tree and ensemble methods are generally appropriate for small- to medium-sized tabular datasets, while DL is better suited to images, spectra, and time-series signals, provided that data volume is adequate. Hydration kinetics, pore connectivity, fiber bridging, and penetrant transport can be encoded for cementitious materials; viscoelasticity, diffusion barriers, interfacial adhesion, and aging kinetics can be incorporated for asphalt and coatings. Similar formulations should not be divided between training and test sets when doing so would cause information leakage. Independent testing, uncertainty intervals, and applicability domains should be reported. SHAP and PDP results should be checked against material mechanisms, and statistical associations should not be presented as causal relationships without additional evidence.

Third, research should move from single-property prediction to multi-property optimization and closed-loop materials design. A progressive framework can be established comprising a standardized database, an interpretable predictive model, physics-constrained multi-objective optimization, and active-learning-guided experiments. Material, processing, structure, service-environment, and property fields should first be harmonized; single- or multi-task models should then be selected according to the data type. Mechanisms such as cement hydration, asphalt aging, or coating-barrier transport can subsequently constrain NSGA-II or BO searches for Pareto-optimal solutions that balance mechanical performance, durability, functionality, and cost. High-information-gain experiments should finally validate candidate designs and update the model. Hierarchical models or transfer learning may separate shared descriptors from matrix-specific features in cross-matrix studies.

Finally, engineering implementation requires a transition from internal cross-validation to independent, cross-matrix, and long-term service validation. Models should be externally tested across raw-material sources, equipment, laboratories, and engineering scenarios, and their applicability domains and prediction intervals should be stated explicitly. Cementitious-material studies should address field mixtures, scale effects, and long-term durability; asphalt, coating, and thermal-management studies should examine hygrothermal cycling, ultraviolet or thermo-oxidative aging, interfacial failure, and functional decay. Emerging materials such as CDs also require assessment of long-term dispersion stability, matrix compatibility, and potential release. Coordinated development of standardized databases, physics-informed models, automated experimentation, and engineering demonstrations may ultimately shift nano-modified construction materials from empirical formulation toward reliable closed-loop intelligent optimization.

## Figures and Tables

**Table 1:** Keyword rankings within the screened ML–nanomaterial–construction domain: Part A, top 10 keywords by occurrence; Part B, top five keywords by betweenness centrality. “Year” is the first year in which the keyword appeared in the analyzed dataset. The table preserves the raw CiteSpace node labels and frequencies; singular/plural and synonymous forms are intentionally displayed separately because post hoc merging would change node frequencies and centralities without a complete rerun of the network analysis.

Part A: Keyword	Occurrence	Centrality	First Year
machine learning	258	0.05	2020
carbon nanotubes	184	0.04	2015
mechanical property	172	0.07	2013
artificial neural network	159	0.22	2013
performance	157	0.08	2017
graphene oxide	134	0.07	2017
composites	131	0.07	2013
compressive strength	115	0.05	2012
behavior	112	0.08	2013
nano silica	95	0.03	2013
Part B: artificial neural network	159	0.22	2013
artificial neural networks	45	0.13	2012
mechanical properties	45	0.11	2013
nanoparticles	72	0.1	2012
neural network	42	0.1	2013

**Table 2:** Top 25 keywords with the strongest keyword bursts. Red segments indicate active burst periods, whereas cyan segments represent the remaining observation period.

Keywords	Strength	Begin	End	Jan. 2012–Jul. 2026
central composite design	6.23	2013	2020	▂ ▃▃▃▃▃▃▃▃ ▂▂▂▂▂▂
experimental design	3.6	2013	2018	▂ ▃▃▃▃▃▃ ▂▂▂▂▂▂▂▂
artificial neural network	14.24	2014	2019	▂ ▂ ▃▃▃▃▃▃ ▂▂▂▂▂▂▂
aqueous solution	9.2	2015	2020	▂▂▂ ▃▃▃▃▃▃ ▂▂▂▂▂▂
activated carbon	8.84	2015	2019	▂▂▂ ▃▃▃▃▃ ▂▂▂▂▂▂▂
methylene blue	9	2016	2021	▂▂▂▂ ▃▃▃▃▃▃ ▂▂▂▂▂
response surface methodology	8.52	2016	2019	▂ ▂▂▂ ▃▃▃▃ ▂▂▂▂▂▂▂
waste water	4.92	2017	2020	▂▂▂▂▂ ▃▃▃▃ ▂▂▂▂▂▂
genetic algorithm	4.87	2017	2022	▂▂▂▂▂ ▃▃▃▃▃▃ ▂▂▂▂
solid phase extraction	3.81	2017	2018	▂▂▂▂▂ ▃▃ ▂▂▂▂▂▂▂▂
reduced graphene oxide	4.27	2018	2020	▂▂▂▂▂▂ ▃▃▃ ▂▂▂▂▂▂
GO	5.89	2020	2023	▂▂▂▂▂▂▂▂ ▃▃▃▃ ▂▂▂
resistance	4.7	2020	2021	▂▂▂▂▂▂▂▂ ▃▃ ▂▂▂▂▂
sensitivity analysis	4.2	2020	2023	▂▂▂▂▂▂▂▂ ▃▃▃▃ ▂▂▂
high performance concrete	5.45	2021	2023	▂▂▂▂▂▂▂▂▂ ▃▃▃ ▂▂▂
model	4.56	2021	2022	▂▂▂▂▂▂▂ ▂▂ ▃▃ ▂▂▂▂
walled carbon nanotubes	3.77	2021	2022	▂▂▂▂▂▂ ▂▂▂ ▃▃ ▂▂▂▂
classification	3.08	2021	2023	▂▂▂▂▂▂▂▂▂ ▃▃▃ ▂▂▂
regression	4.8	2022	2023	▂▂▂▂▂▂▂▂▂▂ ▃▃ ▂▂▂
mechanism	3.47	2022	2024	▂▂▂▂▂▂▂▂▂▂ ▃▃▃ ▂▂
strain	3.16	2022	2024	▂▂▂▂▂▂▂▂▂▂ ▃▃▃ ▂▂
machine learning	22.57	2024	2026	▂▂▂▂▂▂▂▂ ▂▂▂▂ ▃▃▃
cement	4.77	2024	2026	▂ ▂▂▂▂▂▂▂▂▂▂▂ ▃▃▃
oxide	3.7	2024	2026	▂▂▂▂▂▂▂▂▂▂▂▂ ▃▃▃
nanocomposite	3.11	2024	2026	▂▂▂▂▂▂▂ ▂▂▂▂▂ ▃▃▃

**Table 3:** Comparative assessment of representative ML studies discussed in Section 3. NR, not reported in the reviewed article.

System	N	Task	Model(s)	Validation	Reported Result	External	Experiment
Nano-SiO_2_ concrete [6]	1143	Strength prediction	ANN/RF/SVM	Train–test	R^2^: RF 0.93; ANN 0.92	No	No
Nano-SiO_2_/RHA material [40]	Small; NR	Strength + durability	RF/ANN	Train–test	R^2^ 0.858–0.990	No	No
Nano-SiO_2_ concrete [41]	NR	Prediction + multi-objective search	XGBoost + NSGA-II	Train–test	R^2^ 0.99	No	NR
Fiber/nano-SiO_2_ concrete [42]	NR	Strength prediction	Ensembles + SHAP	Train–test	R^2^ 0.92	No	No
RAC + nano-SiO_2_ [43]	420/240	Strength + acid resistance	XGBoost/RF	Train–test	R^2^ 0.995/0.909	No	No
ZrO_2_/MWCNT composite [44]	301	Strength + formulation screening	XGBoost	Train–test	R^2^ 0.990	No	NR
CNT cement composites [45]	429	Strength + parameter optimization	PSO-XGBoost + SHAP	Train–test	R^2^ 0.91	No	No
GO concrete [46]	NR	Strength prediction	MLR/KNN/RF/XGBoost	Train–test	R^2^ 0.981	No	Experimental consistency
GO concrete [47]	367	Strength prediction	Metaheuristic-RF + SHAP	Train–test	R^2^ 0.93	No	No
GO/silica-fume concrete [48]	750	Strength prediction	Eight algorithms	Train–test	R^2^ 0.776; RMSE 2.47 MPa	No	No
GO-based adsorbents [49]	100,000	Transferable adsorption workflow	DL/ANN	Train–test	R^2^ 0.988	No	No
Nano-ZnO/SBR asphalt [50]	NR	Aging/rheology prediction	XGBoost/RF + SHAP	Train–test	R^2^ > 0.90	No	No
CNT/GO nanocoatings [51]	NR	Heat-transfer prediction	SVR/LSSVR/GPR/ELM/ANN	NR	GPR best; value NR	No	No
CD corrosion inhibitor [52]	102	Prediction + synthesis optimization	RF + GA	10-fold CV	Predicted 94.6%; measured 92.3%	No	Yes

**Table 4:** Practical guidance for selecting ML methods in nano-modified construction-material research.

Method	Best-Suited Data	Strengths	Limitations	Recommended Role
Linear/RSM	Small, structured; baseline	Transparent; low variance	Misses strong nonlinearity	Baseline and screening
SVR	Small-to-medium tabular	Effective in high-dimensional small samples	Kernel-sensitive; limited scaling	Strength/durability prediction
RF	Small-to-medium heterogeneous tabular	Robust; nonlinear; feature importance	Piecewise extrapolation; correlated-feature bias	Benchmarking and interpretation
XGBoost/CatBoost	Medium tabular	High accuracy; interactions; missing-data tolerance	Tuning and leakage sensitivity	Property prediction and surrogate modeling
ANN/ELM	Medium-to-large nonlinear tabular	Flexible multi-output mapping	Data hungry; variable stability	Multi-property prediction
CNN/DNN/RNN	Images, spectra, sequences	Automatic representation learning	Large data/computation; opacity	Segmentation, spectra, sensing
GA/PSO/NSGA-II	Search over validated surrogate	Flexible constraints and Pareto search	No guarantee beyond surrogate domain	Formulation optimization
BO/active learning	Costly sequential experiments	Sample-efficient; uncertainty-aware	More difficult for many objectives	Closed-loop experimental design

**Table 5:** Principal limitations, their consequences, and priority research needs.

Limitation	Consequence	Priority Need
Small and imbalanced datasets	Unstable ranking; optimistic error	Open matrix-aware datasets; learning curves; uncertainty intervals
Incomplete nano-descriptors	Material labels confound source and chemistry	Report size, surface area, functionality, defects, dispersion energy
Random-split leakage	Inflated generalization	Grouped, source-wise, temporal, and external validation
Metric inconsistency	R^2^ values cannot be compared directly	Report MAE/RMSE/R^2^ with units, baselines, and intervals
Weak physical consistency	Accurate but implausible trends	Physics-informed features, constraints, and mechanism checks
Prediction–optimization conflation	Unverified “optimum” formulations	Separate stages; define bounds/objectives; experimental verification
Sparse durability/function data	Short-term strength dominates	Long-duration, cyclic, multi-task, and service-condition datasets
Limited cross-matrix transfer	Poor portability	Applicability domains, hierarchical models, and transfer learning

## Data Availability

Bibliographic records were retrieved from the Web of Science Core Collection and are subject to the database provider’s access conditions. The search equation, eligibility criteria, and analytical settings used in this review are reported in Section 2.1. Processed bibliometric and study-extraction data are available from the corresponding authors upon reasonable request.
